# Mechanochemically Synthesized Covalent Organic Framework Effectively Captures PFAS Contaminants

**DOI:** 10.1002/smll.202509275

**Published:** 2025-09-18

**Authors:** Maroof Arshadul Hoque, Thomas Sommerfeld, Jan Lisec, Prasenjit Das, Carsten Prinz, Christian Heinekamp, Tomislav Stolar, Martin Etter, David Rosenberger, Janine George, Biswajit Bhattacharya, Franziska Emmerling

**Affiliations:** ^1^ BAM Federal Institute for Materials Research and Testing Richard‐Willstätter‐Str. 11 12489 Berlin Germany; ^2^ Department of Chemistry Functional Materials Technical University Berlin Hardenbergstraße 40 10623 Berlin Germany; ^3^ Department of Chemistry Humboldt University Berlin Brook‐Taylor‐Straße 2 12489 Berlin Germany; ^4^ Deutsches Elektronen‐Synchrotron (DESY) Notkestraße 85 22607 Hamburg Germany; ^5^ University of Jena Institute of Condensed Matter Theory and Optics Max‐Wien‐Platz 1 07743 Jena Germany

**Keywords:** adsorption, COF, in situ, mechanochemistry, PFAS

## Abstract

Per‐ and polyfluoroalkyl substances (PFAS) are persistent environmental contaminants that pose significant health risks, prompting urgent efforts to develop effective removal methods and adsorbers. Covalent organic frameworks (COFs) are metal‐free adsorbers with high stability and tunable porosity. A highly crystalline COF is synthesised mechanochemically using 1,3,5‐tris(4‐aminophenyl)benzene (TAPB) and 1,3,5‐triformylbenzene (TFB). The formation dynamics are monitored in real time with time‐resolved in situ synchrotron X‐ray diffraction. The TAPB‐TFB COF demonstrates good efficiency in eliminating PFAS from water. Perfluorooctanoic acid (PFOA) and perfluorooctanesulfonic acid (PFOS) are effectively extracted, and most of the adsorption occurred within the first 10 min. Additionally, X‐ray photoelectron spectroscopy, Fourier transform infrared spectroscopy, and DFT calculations are employed to elucidate the molecular interactions between PFAS and the COF framework. The rapid and efficient removal of PFAS makes TAPB‐TFB COF a promising material for water treatment applications.

## Graphical abstract

PFAS are persistent pollutants posing health risks. For PFAS removal, covalent organic frameworks (COFs) offer a metal‐free, stable alternative. A TAPB‐TFB COF is synthesized mechanochemically and monitored via time‐resolved in situ synchrotron X‐ray diffraction. It efficiently removes the majority of PFOA and PFOS in water within 10 min, highlighting its potential for water treatment applications.

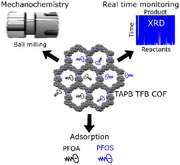

## 1 Introduction

Per‐ and polyfluoroalkyl substances (PFAS) have attracted global attention due to their toxicological profile, bioaccumulative nature, and ubiquitous distribution and persistence in the environment.^[^ ^1^, ^2^, ^3^ ^]^ The exceptionally strong carbon─fluorine bond (≈485 kJ mol^−1^)^[^ ^4^ ^]^ makes PFAS resistant to biological degradation and conventional waste treatment processes, earning them the name “forever chemicals”.^[^ ^1^, ^2^, ^5^ ^]^ These molecules have found their way into soil^[^ ^5^ ^]^ and eventually into drinking water.^[^ ^6^, ^7^ ^]^ Among the different PFAS molecules, perfluorooctanoic acid (PFOA) and perfluorooctanesulphonic acid (PFOS) are the most widespread and biopersistent.^[^ ^8^, ^9^ ^]^ In 2024, the International Agency for Research on Cancer (IARC) reclassified PFOA as “carcinogenic to humans” (Group 1), with PFOS remaining “possibly carcinogenic to humans” (Group 2B).^[^ ^10^ ^]^ Therefore, the removal of these contaminants from drinking water is crucial.^[^ ^11^, ^12^ ^]^

A number of solid adsorbents, such as activated carbon,^[^ ^13^, ^14^ ^]^ anion‐exchange resins,^[^ ^15^, ^16^ ^]^ porous organic polymers,^[^ ^17^, ^18^ ^]^ metal oxides,^[^ ^19^, ^20^ ^]^ and metal‐organic frameworks (MOFs)^[^ ^21^, ^22^ ^]^ have been tested for PFAS removal from contaminated water. Over the past few years, MOFs have shown significant promise in this regard due to their high surface area and tunable pore structures.^[^ ^21^, ^22^ ^]^ However, these materials often have limitations in terms of selectivity, uptake capacity, or stability.^[^ ^23^ ^]^ Despite their advances, MOFs suffer from instability in aqueous environments, typically going along with metal‐linker bond breakage.^[^ ^24^ ^]^ Recently, covalent organic frameworks (COFs) have emerged as a promising alternative for PFAS adsorption.^[^ ^25^, ^26^, ^27^, ^28^ ^]^ These materials are characterised by high porosity, large internal surface area, and the incorporation of functional groups.^[^ ^29^, ^30^ ^]^ In addition, the covalent bonding in these structures results in high stability. Therefore, COFs are better suited as adsorbent materials.^[^ ^25^, ^26^, ^27^, ^28^ ^]^

To date, the application of COFs has been limited by drawbacks associated with the conventional solvothermal synthesis method.^[^ ^31^ ^]^ This method relies on small‐batch syntheses, dissolving precursors in toxic organic solvents and subjecting them to precise pressure and temperature conditions. In addition, extended reaction times and high energy consumption are environmental concerns.^[^ ^32^, ^33^, ^34^ ^]^ In this regard, mechanochemistry,^[^ ^35^, ^36^ ^]^ an alternative synthesis method driven by mechanical forces like compression and shear, offers key advantages such as reduced reaction times, mild conditions, solvent‐free processes, and high scalability.^[^ ^32^, ^33^, ^34^, ^37^ ^]^ Recently, mechanochemistry has been used for the synthesis of porous COFs for different functional applications.^[^ ^38^, ^39^, ^40^ ^]^ To our knowledge, no mechanochemically synthesized COFs have been used as PFAS adsorbents so far.

Here, we report the mechanochemical synthesis of a 2D imine COF (TAPB‐TFB COF) by the reaction of TAPB and TFB. Although this COF was already synthesized by solvent chemistry, mechanochemical synthesis and its adsorption of PFAS has not been explored.^[^ ^41^ ^]^ The pore size (≈1.6 nm) of the TAPB‐TFB COF exceeds the dimensions of key PFAS molecules such as PFOA and PFOS (≈1–1.2 nm).^[^ ^42^, ^43^ ^]^ This feature, combined with its large open channels, makes it suitable for adsorption. The mechanochemical synthetic route for the TAPB‐TFB COF was systematically optimised regarding liquid additive, mechanical impact, and reaction time. Time‐resolved in situ synchrotron X‐ray diffraction was used to gain insight into the mechanochemical reaction pathways. The COF was fully characterised by various analytical techniques, and its affinity for the removal of PFOA and PFOS from water was evaluated. The TAPB‐TFB COF appears to be a promising material for the efficient removal of PFAS from water.

## 2 Results and Discussion

**Figure 1:**
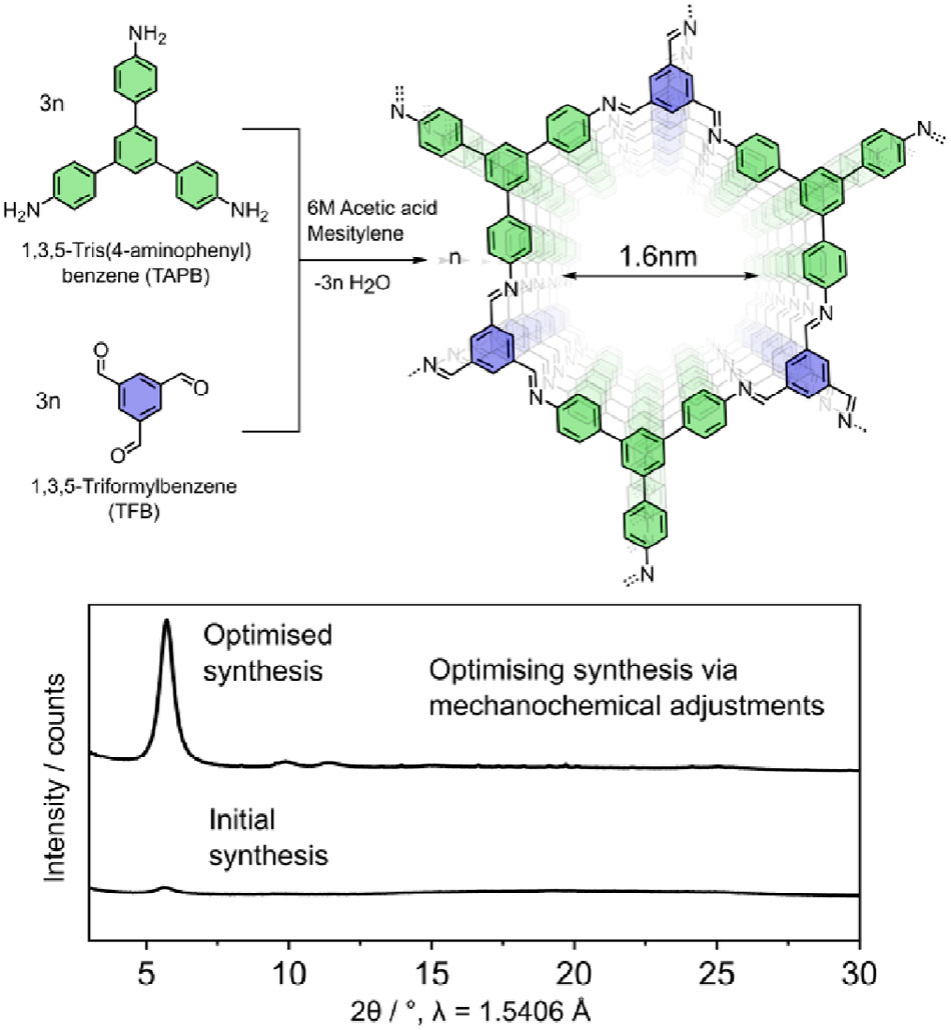
Scheme illustrating the imine condensation reaction between TAPB and TFB to form TAPB‐TFB COF. The synthesis of the COF was optimised by systematically adjusting various mechanochemical parameters, including reagent quantities, liquid additive volume, reaction vessel type, number of milling balls, reaction time, and milling frequency.

**Figure 2:**
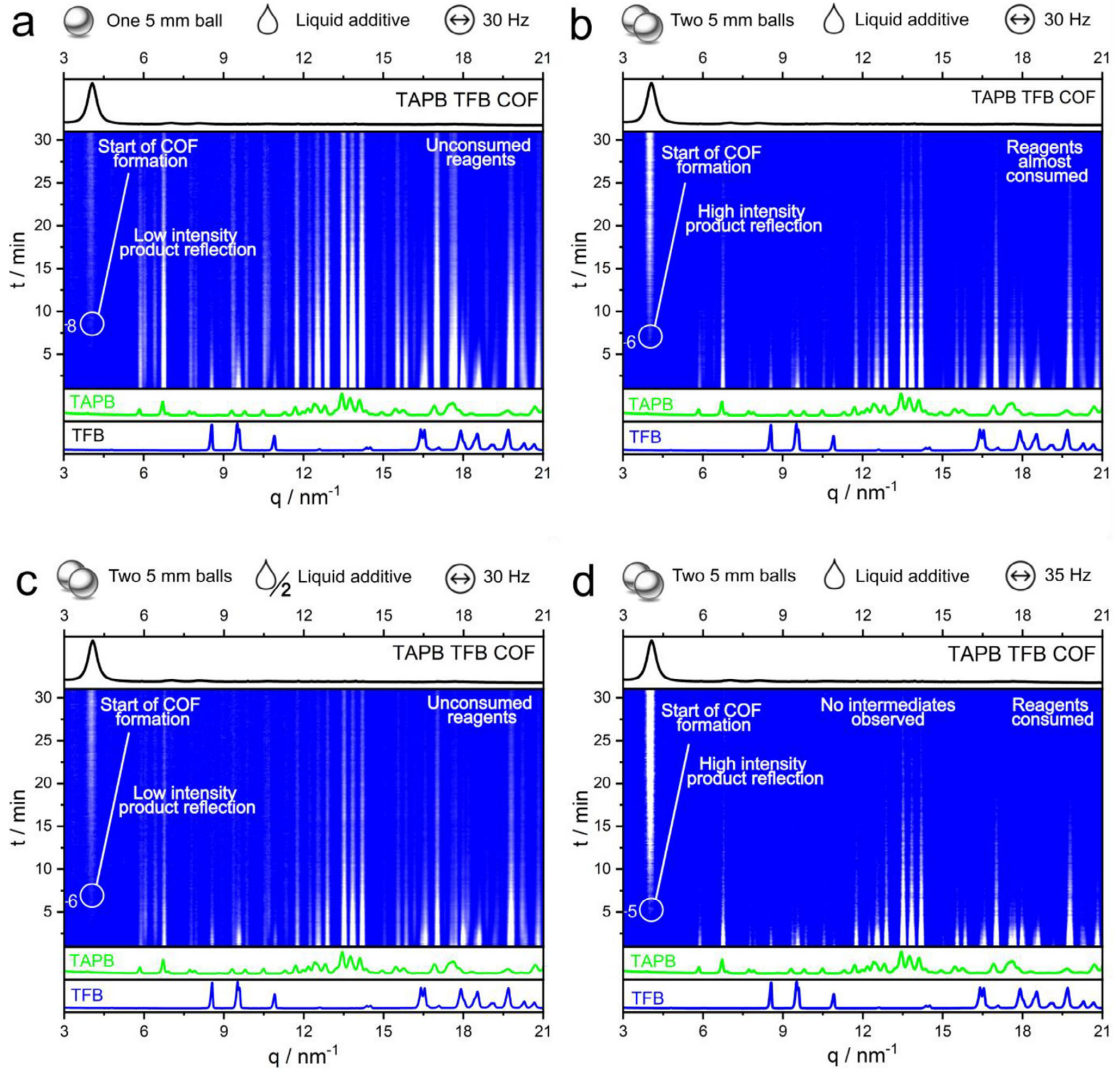
Overview of time‐resolved in situ synchrotron XRD data for the mechanochemical synthesis of TAPB‐TFB COF under different conditions. The bottom panel depicts the powder XRD pattern of the reactants, while the top panel depicts the powder XRD pattern of the product COF, both in q (nm^−1^). “q” represents the scattering vector, and it quantifies the momentum transfer in reciprocal space during scattering experiments. When analyzing synchrotron data, q is preferred over 2θ because q provides a standardized measure of the momentum transfer that is independent of the specific X‐ray wavelength used. a) Synthesis using 0.5 mmol of each reagent, 112.5 µL mesitylene, and 150 µL 6 m acetic acid with one stainless‐steel ball (5 mm in diameter) in a 10 mL jar, milled at 30 Hz for 90 min. b) Same parameters as reaction a, but with two milling balls instead of one. c) Conditions as in reaction b, but with half the volume of liquid additive. d) Conditions as in reaction b, but with an increased milling frequency of 35 Hz. The reaction conditions chosen in b and d resulted in bright diffraction peaks of the final product.

Mechanochemical synthesis was used to produce TAPB‐TFB COF by imine condensation of TAPB and TFB (**Figure** **1**; Figure S1, Supporting Information). Different milling parameters were varied to optimise yield and crystallinity of the final product, including reagent amounts, type and volume of liquid additive, intensity of mechanical impact, size and type of reaction vessel, and reaction time. The successful synthesis was achieved by reacting the precursors in a 10 mL stainless steel jar and adding a mixture of mesitylene and acetic acid. The reaction was performed at a frequency of 30 Hz for 90 min using a single 5 mm stainless steel ball (Figure S1, Supporting Information). The formation of the COF was confirmed by powder X‐ray diffraction (XRD), which showed a weak reflection, i.e., low crystallinity at 5.6° 2θ (λ = 1.5406 Å) (Figure 1; Figure S2, Supporting Information). Crystallinity is crucial for adsorption applications, as higher crystallinity results in a more ordered framework with well‐defined pores. This is essential for effective adsorption of guest molecules. To understand the formation process and improve the crystallinity of TAPB‐TFB COF, we used time‐resolved in situ synchrotron X‐ray diffraction.^[^ ^44^ ^]^ This technique provided insights into the mechanochemical synthesis dynamics, allowing the observation of structural evolution in real time. For real‐time monitoring, transparent PMMA jars were used instead of stainless‐steel jars to allow X‐rays to pass through without significant attenuation of the beam. Initially, the reaction conditions used were identical to those optimised in the stainless‐steel jars. A systematic series of mechanochemical experiments was conducted to investigate the influence of key reaction parameters (**Figure** **2**).

As shown in Figure 2a, when milled with a single 5 mm ball, the reflections assigned to the COF began to emerge within 8–10 min. This is indicated by the appearance and increase in intensity of a reflection at a low q value of 4.1 nm^−1^, corresponding to the COF's characteristic reflection. Initially, strong reflections of the reactants, TAPB and TFB, were observed, but these diminished over time. Notably, the COF formation occurred directly, without the formation of any intermediate phases.

In a subsequent reaction, two milling balls were used. Using equimolar amounts of each reactant with mesitylene and 6 m acetic acid, the formation of COF was observed earlier at around 7 min (Figure 2b). The use of a second ball also resulted in a more pronounced increase in intensity as the reaction progressed. To optimise the TAPB‐TFB COF synthesis, we systematically varied the reaction parameters while maintaining the conditions as shown in Figure 2b. Halving the liquid additive volume (Figure 2c) or reducing the milling frequency to 25 Hz (Figure S3, Supporting Information) slowed product formation and weakened COF reflection intensity. Conversely, increasing the frequency to 35 Hz (Figure 2d) accelerated reaction kinetics and increased reflection intensity. The use of two 5 mm balls increased the impact energy and accelerated the reaction. However, excessive mechanical force, either using more balls or prolonged reaction time, could potentially damage the COF framework (Figures S4 and S5, Supporting Information). The volume of liquid additive proved to be critical. Reduced amounts resulted in slower rates, while excessive amounts were likely to dampen impact energy. Additionally, since water is the main byproduct of imine condensation, we examined whether its addition affects COF formation. XRD analysis showed that the addition of water led to weaker COF peaks and unreacted starting materials. Using only water as a liquid additive resulted in no COF formation (Figures S6 and S7, Supporting Information). Using these insights, we optimised laboratory conditions for crystalline TAPB‐TFB COFs. Optimal parameters included equimolar amounts (0.5 mmol) of TAPB and TFB, 112.5 µL mesitylene, 150 µL 6 m acetic acid, and a milling time of 90 min at 30 Hz in a horizontal ball mill. Soxhlet extraction with methanol as the washing solvent was employed for the washing step. The effectiveness of our optimised synthesis was confirmed through a comparison of the XRD patterns of TAPB‐TFB COFs synthesized via mechanochemical and solvothermal methods, which exhibited a high degree of agreement (Figure S8, Supporting Information).

**Figure 3:**
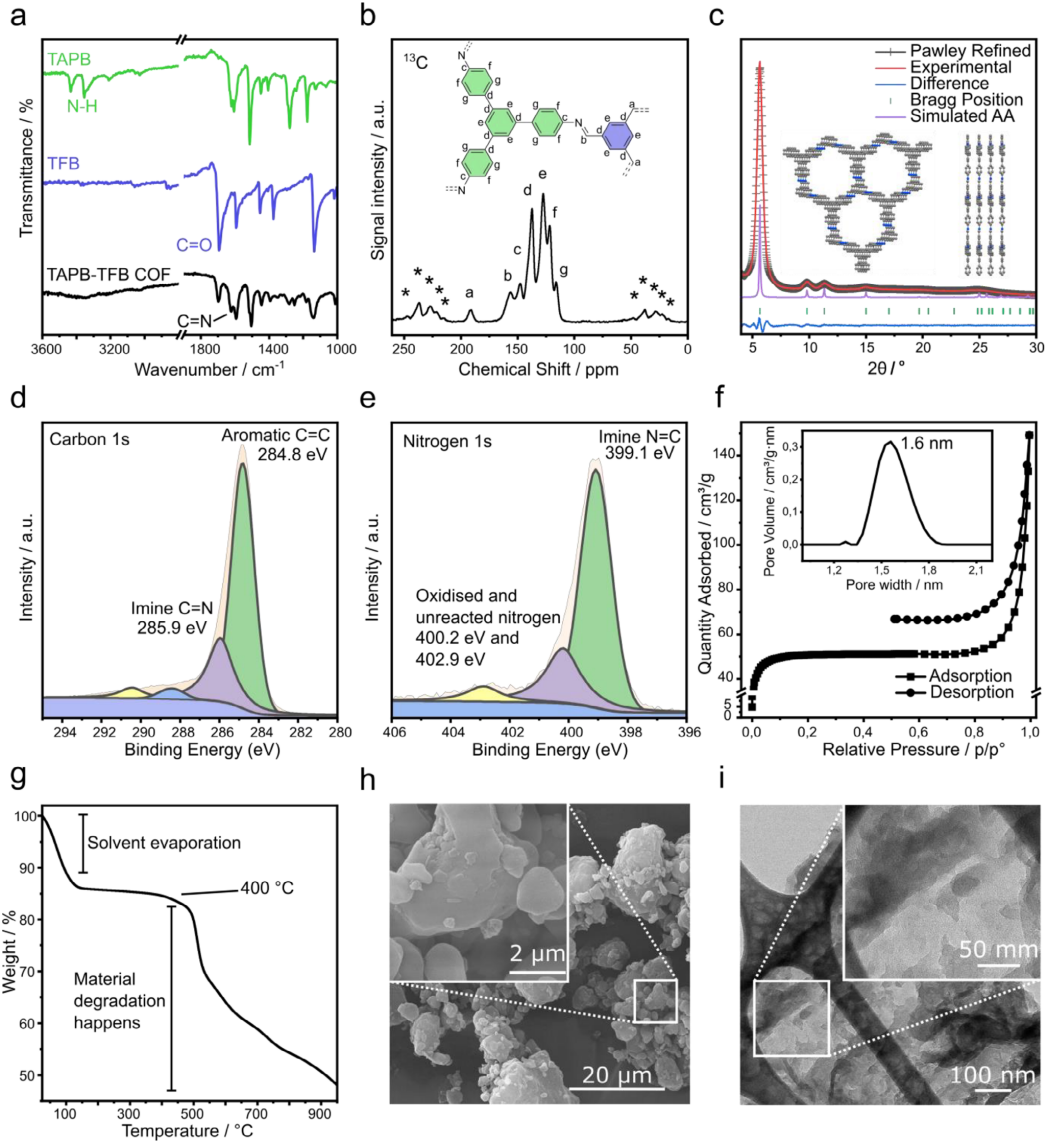
a) Comparison of the FT–IR spectra of the reactants and the product COF. The disappearance of the N─H bond, along with the appearance of the C═N bond, confirms the formation of the TAPB‐TFB COF. b) The ^13^C solid‐state magic angle spinning nuclear magnetic resonance (NMR) spectrum of the TAPB‐TFB COF confirms the presence of a C═N bond, thereby verifying the imine condensation reaction between TAPB and TFB. The stars in the figure refer to repeating rotational sidebands that occur in solid‐state NMR. c) Pawley refinement of the XRD pattern of mechanochemically synthesised TAPB‐TFB COF. d) Carbon 1s X‐ray photoelectron spectroscopy (XPS) spectrum showing imine C═N and aromatic C═C bond binding energies. e) Nitrogen 1s XPS spectrum showing imine C═N and oxidized and unreacted amine bond binding energies. f) Adsorption isotherm of the TAPB‐TFB COF performed with nitrogen at 77 K, along with a DFT pore size distribution plot showing a pore size of 1.6 nm. g) Thermogravimetric analysis (TGA) of TAPB‐TFB COF showing the stability of the material till 400 °C. h) An SEM image showing the agglomeration of small COF particles. i) TEM image showing a single layer of COF.

TAPB‐TFB COF formation proceeds via imine condensation between the amine and carbaldehyde groups of TAPB and TFB. This process was conclusively confirmed by Fourier transform infrared (FT–IR) spectroscopy (**Figure** **3**a). The FT–IR spectrum of TAPB shows characteristic bands at 3433 cm^−1^ for N─H asymmetric stretching frequency and at 3350 cm^−1^ for N─H symmetric stretching frequency, while the TFB spectrum shows a strong C═O stretching band at 1690 cm^−1^. In the spectrum of the TAPB‐TFB COF, we observed the disappearance of the N─H stretching vibrations and the formation of a medium intensity band at 1621 cm^−1^, stemming from the imine bond formation,^[^ ^41^ ^]^ indicative of a successful COF formation by mechanochemistry. A band at 1690 cm^−1^ was also observed for the COF, which could imply the presence of unreacted aldehyde groups.

^13^C solid‐state magic‐angle spinning NMR spectroscopy provided further evidence for the TAPB‐TFB COF structure (Figure 3b). The spectrum revealed characteristic signals at 155.9 ppm, originating from the newly formed imine carbon C═N. The signal at 147.5 ppm was assigned to the carbon atom in TAPB directly connected to the imine bond. Aromatic carbons within the COF scaffold were identified by signals at 127.2, 121.5, and 115.9 ppm. As already evident from the FT–IR results, a distinct peak at 190.6 ppm indicated the presence of C═O bonds, likely from unreacted aldehyde groups at framework layer edges.^[^ ^41^ ^]^

PXRD analysis confirmed the crystallinity of the TAPB‐TFB COF (Figure 3c). The pattern showed a prominent reflection at 2θ = 5.6° (λ = 1.5406 Å) corresponding to the (100) plane. Additional reflections were observed at 9°, 11°, 14.9°, and 25.2° for the (210), (200), (310), and (001) planes, respectively. Based on these data, we proposed a 2D structure with inclined AA stacking in the trigonal P3 space group (Figure S9, Supporting Information). Pawley refinement (Figure 3c) confirmed excellent agreement between experimental and simulated patterns (R_p_ = 2.82%, R_wp_ = 3.66%). The *π*–*π* stacking distance between the layers was calculated to be 3.54 Å. X‐ray photoelectron spectroscopy (XPS) analysis of TAPB‐TFB COF confirmed the formation of TAPB‐TFB COF. The high‐resolution carbon 1s spectrum of TAPB‐TFB COF (Figure 3d) exhibited distinct peaks at 284.8 and 285.9 eV, corresponding to aromatic C═C and imine C═N bonds, respectively. Similarly, the nitrogen 1s spectrum (Figure 3e) displayed peaks at 399.1, 400.2, and 402.9 eV. The peak at 399.1 eV was assigned to imine C═N, while the other two were assigned to unreacted and dangling amine groups at the edges of the framework.^[^ ^41^ ^]^

Nitrogen adsorption‐desorption measurements revealed a mixture of type I and type II sorption isotherms for the TAPB‐TFB COF, with significant uptake at low pressures (P/P_o_ = 0–0.03). The calculated BET surface area was 202.19 m^2^ g^−1^, with a pore volume of 0.07 cm^3^ g^−1^. The DFT pore width was calculated to be 1.6 nm (Figure 3f; Figures S10 and S11, Supporting Information). TGA showed high thermal stability up to 400 °C, with an initial 15% weight loss at 84 °C attributed to solvent evaporation from the COF pores (Figure 3g). SEM analysis revealed small, irregularly shaped particles, probably resulting from high mechanical stress during synthesis (Figure 3h; Figure S12, Supporting Information). High magnification TEM imaging revealed layered structures resembling crumpled paper (Figure 3i; Figure S13, Supporting Information).

The 2D layered structure and porosity of the TABP‐TFB COF make it a promising candidate for PFAS removal applications. To evaluate the adsorption capacity, systematic studies were conducted with two prevalent PFAS: PFOA and PFOS. Prior to tests, stability assessments were conducted to ensure the COF's structural integrity in both pure water and water containing PFAS. The COF was subjected to agitation for seven days in water and in a solution of water with PFOA and PFOS. Post‐exposure analysis by PXRD showed that the COF keeps its crystallinity (Figure S14, Supporting Information), which confirms its chemical stability under the tested conditions. Further experiments show that the TAPB‐TFB COF remains stable under both fairly acidic and highly alkaline conditions (Figure S15, Supporting Information).

**Figure 4:**
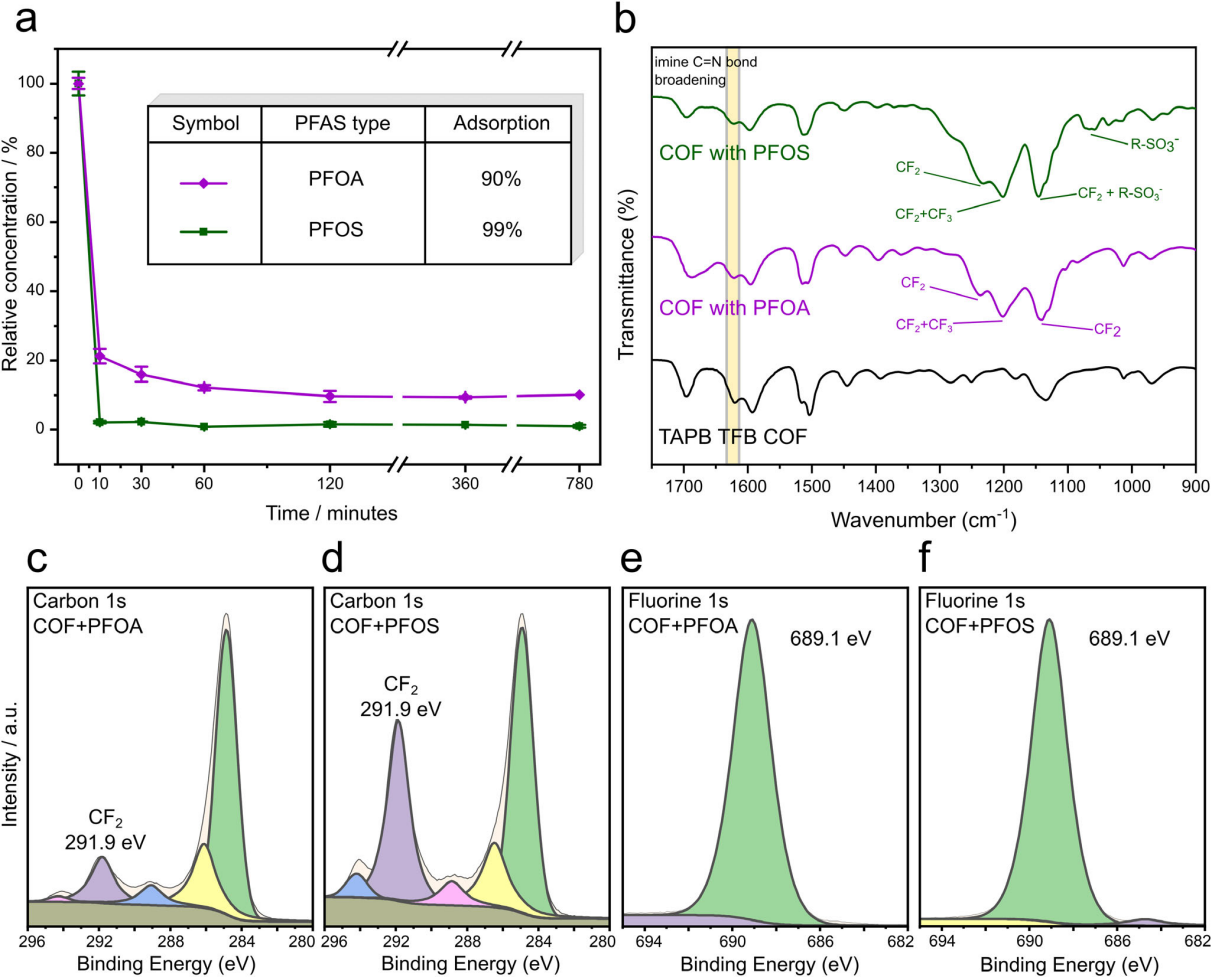
a) Adsorption of PFOA and PFOS by TAPB‐TFB COF over time, showing rapid uptake of 90% PFOA and 99% PFOS of the initial concentration within 13 h, with most pronounced adsorption occurring within the first 10 min. b) FT–IR spectrum before and after adsorption, highlighting additional bands corresponding to PFOA and PFOS and broadening of the imine bond signal. c) Carbon 1s spectrum for PFOA‐adsorbed COF, d) Carbon 1s spectrum for PFOS‐adsorbed COF, e) Fluorine 1s XPS spectrum for PFOA‐adsorbed COF, and f) Fluorine 1s XPS spectrum for PFOS‐adsorbed COF, demonstrating interactions between the COF and the adsorbed species.

The PFAS adsorption efficiency was evaluated by immersing 10 mg of the COF in a solution containing 50 µg L^−1^ of PFOA and PFOS respectively. The residual concentration of PFAS in the solution post‐adsorption was quantified using ultra‐high performance liquid chromatography mass spectrometry (UHPLC‐MS). The adsorption percentages were calculated based on the ratio between the initial and equilibrium concentrations of PFAS. The TAPB‐TFB COF demonstrated 90% adsorption of PFOA and 99% adsorption of PFOS after 13 h of contact. Time‐dependent experiments were conducted at intervals of 10, 30, 60 min, 2, 6, and 13 h. The data revealed that the majority of PFAS uptake occurred within the first 10 min, followed by a plateau, suggesting saturation (**Figure** **4**a). This rapid adsorption is likely driven by the hydrophobic effect and interactions, together with hydrogen bonding.^[^ ^45^, ^46^, ^47^, ^48^ ^]^ Upon contact with the COF, the ionic PFAS molecules quickly migrate into the framework's hydrophobic pores.^[^ ^45^, ^46^ ^]^ Most probably, the imine groups in the COF framework are partially protonated. The anionic head groups of the PFOA and PFOS and the protonated imine are then connected via a hydrogen bond, thereby facilitating their strong attachment to the COF framework.^[^ ^47^, ^48^ ^]^

To investigate the interaction of PFAS with the COF framework and determine the adsorption sites, COF samples were exposed to concentrated PFOA and PFOS solutions, followed by analysis using XPS and FT–IR spectroscopy. These techniques enabled the identification of PFAS incorporation within the framework. XPS analysis before and after PFAS sorption revealed distinct spectral changes, confirming the successful adsorption of PFOA and PFOS onto the COF framework. In the carbon 1s XPS spectra of both PFOA and PFOS adsorbed COF, the imine and aromatic peaks remained unchanged, while the appearance of new peaks at 292 eV indicated the presence of C─F bonds, which is characteristic of PFAS. Further evidence was observed in the fluorine 1s spectra of both adsorbed species, where a prominent peak at 689.1 eV confirmed fluorine‐containing species within the COF. Additionally, the sulfur 1s XPS spectrum of PFOS‐adsorbed COF exhibited two distinct peaks (Figure S16, Supporting Information). One at 168.5 eV, attributed to sulfonate groups, and another at 169.6 eV, indicative of oxidized sulfur species.

Further confirmation of PFAS adsorption was obtained through FT–IR spectroscopy. Comparing the spectra reveals distinct additional peaks for PFAS‐adsorbed COFs. A band at 1201 cm^−1^ is observed for both PFOA and PFOS‐adsorbed COFs, corresponding to CF_2_ and CF_3_ asymmetric vibrations.^[^ ^49^ ^]^ Another band at 1237 cm^−1^, also common to both, is attributed to asymmetric CF_2_ vibrations.^[^ ^49^ ^]^ A key distinction is seen at 1066 cm^−1^, attributed to asymmetric SO_3_^−^ stretching frequency, which is present in the PFOS‐adsorbed COF but absent in the PFOA‐adsorbed COF.^[^ ^49^ ^]^ Furthermore, PFOS‐adsorbed COF exhibits a vibration at 1146 cm^−1^, corresponding to asymmetric CF_2_ + asymmetric SO_3_^−^, while PFOA‐adsorbed COF shows a peak at 1141 cm^−1^, attributed to symmetric CF_2_ vibrations.^[^ ^49^ ^]^ Additionally, the interaction between COF and PFAS is highlighted by the broadening of the signal of the imine bond at 1621 cm^−1^ for the PFAS adsorbed COF compared to the COF before adsorption. This signal supports the hypothesis of a hydrogen bond between the protonated imine and the anionic groups of PFOS and PFOA.

To shed further light on the hydrogen bond, we evaluated its strength using density functional theory (DFT) as implemented in Vienna ab initio simulation package (VASP)^[^ ^50^, ^51^, ^52^ ^]^ at the Perdew‐Burke‐Ernzerhof (PBE) level^[^ ^53^ ^]^ with the corresponding dispersion corrections (D3(BJ))^[^ ^54^ ^]^ in combination with the projector‐augmented wave method.^[^ ^55^ ^]^ To assess the interaction strength, a model system was constructed in which the PFOA with a deprotonated carboxylic acid group interacts with a protonated imine of the COF. The COF framework was fixed, and we optimized the PFOA anion and the hydrogen protonating the imine. Then, the deprotonated carboxylic acid was moved away from the protonated imine, roughly close to the center of the pore, so that the interaction with the COF framework was minimal and the energy was evaluated statically. The resulting computed hydrogen bond strength as an energy difference of the two model systems is ≈−160 kJ mol^−1^, indicating a significant interaction energy. This high static interaction energy is again in line with a likely adsorption of the PFOA anions by the MOF. However, the accurate computation of adsorption enthalpies and heats of adsorption would need to account for various adsorption configurations and the medium in which the adsorption occurs. Nevertheless, our simulations point to a likely adsorption mechanism, and we assume that the mechanism for PFOS will be similar.^[^ ^56^ ^]^ More details on our simulation can be found in figure S17 of supporting information.

These findings collectively demonstrate the presence of PFOA and PFOS in the COF. Overall, the high adsorption efficiency of PFAS by TAPB‐TFB COF highlights its potential as an effective adsorbent for PFAS removal.

## 3 Conclusion

In view of the pressing need to address PFAS contamination for a safer and more sustainable environment, we have successfully synthesised TAPB‐TFB COF via mechanochemical reaction and demonstrated adsorption capabilities for PFOA and PFOS from aqueous solutions. Our comprehensive characterisation, including time‐resolved in situ synchrotron XRD, FT–IR, NMR, and XPS, confirmed the formation of a crystalline COF structure with a BET surface area of 202 m^2^ g^−1^ and a pore size of 1.6 nm. The TAPB‐TFB COF exhibited good PFAS removal performance, achieving 90% PFOA and 99% PFOS adsorption of their initial concentration, with the majority occurring in the first 10 min. The rapid and efficient adsorption of PFAS by the COF was evaluated using XPS and FT–IR spectroscopy. DFT calculations further suggest that the adsorption mechanism involves hydrogen bonding interactions between PFAS molecules and the COF framework. Our results indicate that TAPB‐TFB COF, produced using mechanochemistry, is effective in our tests for PFAS remediation. While we focused on this method, other synthesis approaches may also be effective, though these were not part of our study. This approach provides a sustainable and scalable alternative to traditional solvothermal methods. Further exploration of mechanochemical synthesis for other COF systems could lead to advanced functional materials for environmental applications.

## Conflict of Interest

The authors declare no conflict of interest.

## Supporting information



Supporting Information

## Data Availability

The data that support the findings of this study are available from the corresponding author upon reasonable request.
