# Supplementary material for: Mechanochemically Synthesized Covalent Organic Framework Effectively Captures PFAS Contaminants
Source: Small. 2025 Sep 18;21(44):e09275. doi: 10.1002/smll.202509275 (PMC12590536; doi:10.1002/smll.202509275)
Supplement: Supplementary file 1 — Supporting Information [file SMLL-21-e09275-s001.docx]

Supporting Information
©Wiley-VCH 2016
69451 Weinheim, Germany

MECHANOCHEMICALLY SYNTHESIZED COVALENT ORGANIC FRAMEWORK FOR PFAS ADSORPTION

Maroof Arshadul Hoque^a^, Thomas Sommerfeld^a^, Jan Lisec^a^, Prasenjit Das^b^, Carsten Prinz^a^ Christian Heinekamp^a,c^, Tomislav Stolar^a^, Martin Etter^d^, David Rosenberger^a^, Janine George^a,e^ Biswajit Bhattacharya^a*^, Franziska Emmerling^a,c*^

[a] Maroof Arshadul Hoque, Thomas Sommerfeld, Dr. Jan Lisec, Christian Heinekamp, Dr. Tomislav Stolar, Dr. David Rosenberger, Dr. Janine George, Dr. Biswajit Bhattacharya, PD Dr. Franziska Emmerling
BAM Federal Institute for Materials Research and Testing
Richard-Willstätter-Str. 11, 12489 Berlin (Germany)
Email- [biswajit.bhattacharya@bam.de](mailto:biswajit.bhattacharya@bam.de) (BB); [franziska.emmerling@bam.de](mailto:franziska.emmerling@bam.de) (FE)

[b] Dr. Prasenjit Das
Department of Chemistry, Functional Materials,
Technical University Berlin,
Hardenbergstraße 40, 10623 Berlin (Germany)

[c] Christian Heinekamp, PD Dr Franziska Emmerling
Department of Chemistry,
Humboldt University Berlin
Brook-Taylor-Straße 2, 12489 Berlin (Germany)

[d] Dr. Martin Etter
Deutsches Elektronen-Synchrotron (DESY),
Notkestraße 85, 22607 Hamburg, Germany

[e] Dr. Janine George
University of Jena, Institute of Condensed Matter Theory and Optics,

Max-Wien-Platz 1, 07743 Jena, Germany

**Abstract:** Per- and polyfluoroalkyl substances (PFAS) are persistent environmental contaminants that pose significant health risks, prompting urgent efforts to develop effective removal methods and adsorbers. Covalent organic frameworks (COFs) are metal-free adsorbers with high stability and tunable porosity. A highly crystalline COF was synthesised mechanochemically using 1,3,5-tris(4-aminophenyl)benzene (TAPB) and 1,3,5-triformylbenzene (TFB). The formation dynamics were monitored in real time with time resolved *in situ* synchrotron X-ray diffraction. The TAPB-TFB COF demonstrated good efficiency in eliminating PFAS from water. Perfluorooctanoic acid (PFOA) and perfluorooctanesulfonic acid (PFOS) were effectively extracted and most of the adsorption occurred within the first 10 minutes. Additionally, X-ray photoelectron spectroscopy, Fourier transform infrared spectroscopy and DFT calculations were employed to elucidate the molecular interactions between PFAS and the COF framework. The rapid and efficient removal of PFAS makes TAPB-TFB COF a promising material for water treatment applications.

Table of Contents

| **S1. Chemicals** | **S4** |
| --- | --- |
| **S2. Synthesis** | **S4 – S5** |
| **S3. Characterization methods** | **S5 – S8** |
| **S4. Characterization of COF**  ***S4.1 XRD***  ***S4.2* *In situ XRD***  ***S4.3 Influence of impact***  ***S4.4 Effect of water and acetic acid***  ***S4.5 Mechanochemical and solvothermal COF***  ***S4.6 Simulated structure***  ***S4.7 Nitrogen adsorption***  ***S4.8 SEM***  ***S4.9 TEM*** | **S9 – S**  **S9 – S10**  **S11 – S12**  **S12 – S13**  **S13 – S15**  **S16**  **S16 – S17**  **S17 – S20**  **S20**  **S20 – S21** |
| **S5 Stability tests** | **S21 – S23** |
| **S6 Sulphur 1s XPS of PFOS adsorbed COF** | **S24** |
| **S7 DFT and MLIP Simulations** | **S24 – S27** |
| **S8 References** | **S27** |

**S1. Chemicals**

1,3,5-triformylbenzene (TFB) and 1,3,5-tris(4-aminophenyl)benzene (TAPB) were ordered from BLD Pharmatech GmbH and were of 96% and 97% purity, respectively. The chemicals mesitylene (98% purity), acetic acid (99.8% purity) and methanol (99.8% purity) were procured from TH Geyer GmbH und Co. KG. Additionally, methanol (≥99.9% purity) of the PFAS grade for LC-MS was obtained from the same source for utilisation in UHPLC MS measurements. All chemicals were employed in their original state, without undergoing any further purification processes.

**S2. Synthesis**

TAPB (184.49 mg, 0.5 mmol) and TFB (85.12 mg, 0.5 mmol) were added to a 10mL stainless steel mechanochemistry jar with two 5mm stainless steel balls. Mesitylene (112.5 μL) and 6M acetic acid (150 μL) were added to the jar for liquid assisted grinding (LAG). The reaction was performed in a RETSCH vibrating mill MM 400 for 90 minutes with a frequency of 30 Hz. After the reaction was completed, the obtained powder was dried for an hour and washed using a Soxhlet extractor using methanol as the liquid for 3 days. The final powder was dried in an oven at 60 °C for 6 hours.

Prior to optimizing the synthesis protocol, the initial successful synthesis was achieved by reacting 0.07 mmol of each precursor in a 10 mL stainless steel jar, supplemented with 15 µL of mesitylene and 20 µL of 6 M acetic acid. Subsequent improvements were realized by tripling the reaction scale to 0.21 mmol of each precursor, with proportional increases to 45 µL of mesitylene and 60 µL of 6 M acetic acid, while preserving all other reaction parameters (jar size, ball size, frequency, and reaction time) constant (Figure S2). PXRD analysis of the scaled-up reaction revealed enhanced crystallinity; however, the product crystallinity remained inferior to that of solvothermally synthesized TAPB-TFB COF.

Further challenges emerged during synchrotron experiments, as no reflections were detected, even from the reactants, when the same reaction was performed. This issue was attributed to insufficient powder dispersion within the jar, leading to poor interaction with the X-ray beam. To mitigate this, the reaction scale was increased by an additional 2.5-fold, resulting in 0.5 mmol of each reactant, along with 112.5 µL of mesitylene and 150 µL of 6 M acetic acid. This adjustment ensured adequate powder quantity and dispersion, enabling clear reflections in subsequent experiments. This optimized reaction scale was subsequently adopted for all further experiments.

Despite these optimized conditions, a small amorphous hump is observed in the XRD pattern. To address this, a final washing step was necessary to remove these residual materials. Methanol, acetone, and tetrahydrofuran (THF), as well as combinations of these solvents, were evaluated for their effectiveness in the washing process. Among these, methanol was identified as the most effective solvent, based on multiple rounds of filtration and centrifugation. Once methanol was established as the optimal solvent, various filtration techniques were compared, including centrifugation, simple filtration using filter paper, and Soxhlet extraction. Soxhlet extraction yielded the highest crystallinity in the final COF product. Ultimately, the XRD pattern of the mechanochemically synthesized TAPB-TFB COF closely matched that of the solvothermally synthesized TAPB-TFB COF (Figure), confirming the effectiveness of the optimized synthesis and purification protocols.

**S3. Characterization Methods**

***S3.1 Fourier Transform Infrared Spectroscopy (FT-IR)***

The FT-IR spectra were recorded with a Nicolet 670 FT-IR (ThermoFisherScientific GmbH) in attenuated total reflection (ATR), with which "Golden Gate" sample holder was used. After each measurement, the sample holder was cleaned with ethanol and acetone. The background spectrum was measured in air. 32 scans with a resolution of 4 cm^-1^ were recorded per measurement. The spectra were recorded in transmission mode in a spectral range of 4000 – 600 cm^-1^. The instrument was controlled with OMNIC software. All spectra were mathematically processed in the same way.

***S3.2 Nuclear Magnetic Resonance Spectroscopy (NMR)***

Solid-state MAS (magic angle spinning) nuclear magnetic resonance spectra were recorded at a Bruker AVANCE 400 (B_0_=9.4 T) spectrometer at room temperature. The rotor sizes for 1H and 19F were 2.5 mm and for 13C was 4 mm. The contact time was 1 ms and the pulse length was 5 s.

***S3.3 X-ray diffraction (XRD)***

A D8 Advance Diffractometer (Bruker AXS, Karlsruhe, Germany), equipped with an energy dispersive LynxEye XE-T detector, was employed for the purpose of performing powder X-ray diffraction. The measurements were conducted with a copper X-ray source, with a wavelength of 1.54178 Å, without the use of a monochromator. The measurements were conducted in Bragg-Brentano geometry with an X-ray tube power of 40 kV and 40 mA. The measurement parameters were as follows: a step size of 0.02° and a measurement time of 1 s per step. Evaluation of data was done using the software DIFFRAC.EVA and the evaluated data was plotted using the software Origin.

***S3.4 Thermogravimetric analysis / Differential scanning calorimetry (TGA/DSC)***

TGA/DSC measurements were conducted using a METTLER Toledo TGA/DSC 3+ instrument. A quantity of 6.9087 mg of COF was subjected to a thermal analysis, being heated from 25 °C to 950 °C in an open 70 μL aluminium oxide crucible with a nitrogen flow of 80 mL/min. The heating rate was set at a rate of 10 °C per minute. Data was evaluated and plotted using the software Origin.

***S3.5 Scanning electron microscopy (SEM)***

Scanning electron microscope (SEM) was employed for the examination of the samples. The microscope was an FEI XL30 ESEM, equipped with a tungsten cathode, SE/GSED/BSE detectors, high and low vacuum, an energy dispersive X-ray spectrometer SDD Quantax 200, and a cryotransfer system Alto 1000. Prior to analysis, the samples were coated with a thin layer of gold to facilitate the conduction of electricity.

***S3.6 Transmission electron microscopy (TEM)***

Transmission electron microscopy (TEM) imaging was conducted using a Talos F200S Microscope (Thermo Fisher Scientific) operated at an accelerating voltage of 200 kV. This technique involves the transmission of a high-energy electron beam through a specimen to generate detailed images. Images were acquired in TEM mode using a Ceta 16M camera. Specimen preparation involved depositing 10 µL of sample solution (1 mg of sample dissolved in 0.2 mL of water) onto a 3 mm copper grid (lacey, 400 mesh) and allowing it to air dry at room temperature. Imaging and data analysis were performed using Velox software (version 3.3).

***S3.7 Nitrogen adsorption***

Nitrogen adsorption/desorption isotherms were recorded using a Micromeritics TriStar III surface area analyser at 77 K, with the temperature held constant using a liquid nitrogen bath. Prior to analysis, approximately 200 mg of the COF were degassed for 12 hours at 120 °C under a flow of dry nitrogen. A Brunauer, Emmett, and Teller (BET) model was employed to determine the specific surface area (m² g⁻¹), while DFT pore volume plot was utilized to ascertain the pore width. Evaluation of data was done using the sotware MicroActive from Micromeritics Instrument Corporation and later plotted in Origin.

***S3.8 In situ X-ray diffraction***

*In situ* XRD measurements were conducted using an X-ray energy of 60 keV (λ = 0.207 A˚) at the Powder Diffraction and Total Scattering Beamline P02.1 at the Deutsches Elektronen-Synchrotron (DESY), employing a modified IST-636 mixer mill (InSolido Technologies, Croatia, Zagreb) operating at a frequency of up to 35 Hz. The reactions were conducted in bespoke 14 mL X-ray transparent poly(methyl methacrylate) (PMMA) milling jars. The X-ray beam was set to pass through the bottom of the PMMA reaction vessel. Exposure time was set to 10 s. Diffraction data were collected on a PerkinElmer XRD1621 flat-panel detector positioned 1595 mm from the sample, which consisted of an amorphous Si sensor equipped with a CsI scintillator (pixel number: 2048 × 2048, pixel size: 200 × 200 m^2^). To obtain the classic one-dimensional powder XRD pattern, the two-dimensional diffraction images were integrated with the in house developed Python script. 2D time-resolved plots of in situ monitoring data were created in Python and the background of each diffraction pattern was subtracted prior to plotting.

***S3.9 Adsorption tests***

All adsorption tests were conducted using polypropylene or polyethylene vessels to minimize potential contamination. A 30 mL amount of PFAS solution was introduced into a 50 mL polypropylene centrifuge tube, followed by the addition of 10 mg of the synthesized covalent organic framework (COF). The tubes were then mounted on an orbital shaker set to 400 rpm to ensure consistent agitation. At predetermined time intervals, specific tubes were removed, and the suspension was collected using a polyethylene syringe. The samples were filtered through a 0.22 µm polypropylene filter to remove particulate matter. The resulting filtrates were analyzed via ultra-high-performance liquid chromatography coupled with mass spectrometry (UHPLC-MS) to quantify PFAS concentrations.

***S3.10 Ultra High Performance Liquid Chromatography (UHPLC)***

Chromatographic separation was achieved using an Agilent ZORBAX RRHD Eclipse Plus C18 column (3 × 100 mm, 1.8 μm) column installed on an Agilent 1290 Infinity II UHPLC system. A gradient elution was performed with 5 mM ammonium acetate in water (mobile phase A) and methanol (mobile phase B) at 0.4 mL/min with a total run time of 17 minutes and an injection volume of 2 µL. To minimize background PFAS contamination, the Agilent UHPLC system was equipped with a PFC-Free HPLC conversion kit and a PFC delay column to retain potential per- or polyfluorochemical impurities from the mobile phases.

Dynamic multiple reaction monitoring (dMRM) analysis was performed using an Agilent 6495C LC/TQ mass spectrometer with an Agilent jet stream (AJS) ion source operated in negative ionization mode. Data acquisition and processing were conducted using Agilent MassHunter data LC/MS Acquisition software version 10.0 and Quantitative Analysis software version 10.2, respectively. To enable quantification, calibration curves were prepared using the standard PFAC30PAR from Wellington.

***S3.11 X-ray Photoelectron Spectroscopy (XPS)***

X-ray Photoelectron Spectroscopy (XPS) was measured on a K-Alpha™ + X-ray Photoelectron Spectrometer System (Thermo Scientific) with Hemispheric 180 ° dual focus analyzer with 128-channel detector. The X-ray monochromator used micro focused Al-Kα radiation. For the measurement, the powder samples were pressed and loaded on carbon tape, then pasted onto the sample holder for measurement. The data was collected with an X-ray spot size of 400 μm, 20 scans for the survey, and 50 scans for the regions. Data from XPS was evaluated and plotted using the software Origin.

**S4. Characterization of COF**

***S4.1 XRD***

The XRD analysis of the reactants, TAPB and TFB, displayed characteristic peaks, represented by green and blue respectively. Upon undergoing an imine condensation reaction, these reactants form the TAPB TFB COF. In the product XRD, the distinctive peaks from the reactants disappear, and a prominent peak emerges at a low angle, indicating the formation of the COF structure. This observation is consistent with both literature reports^[1]^ and simulations, further confirming the successful synthesis of TAPB-TFB COF.

**
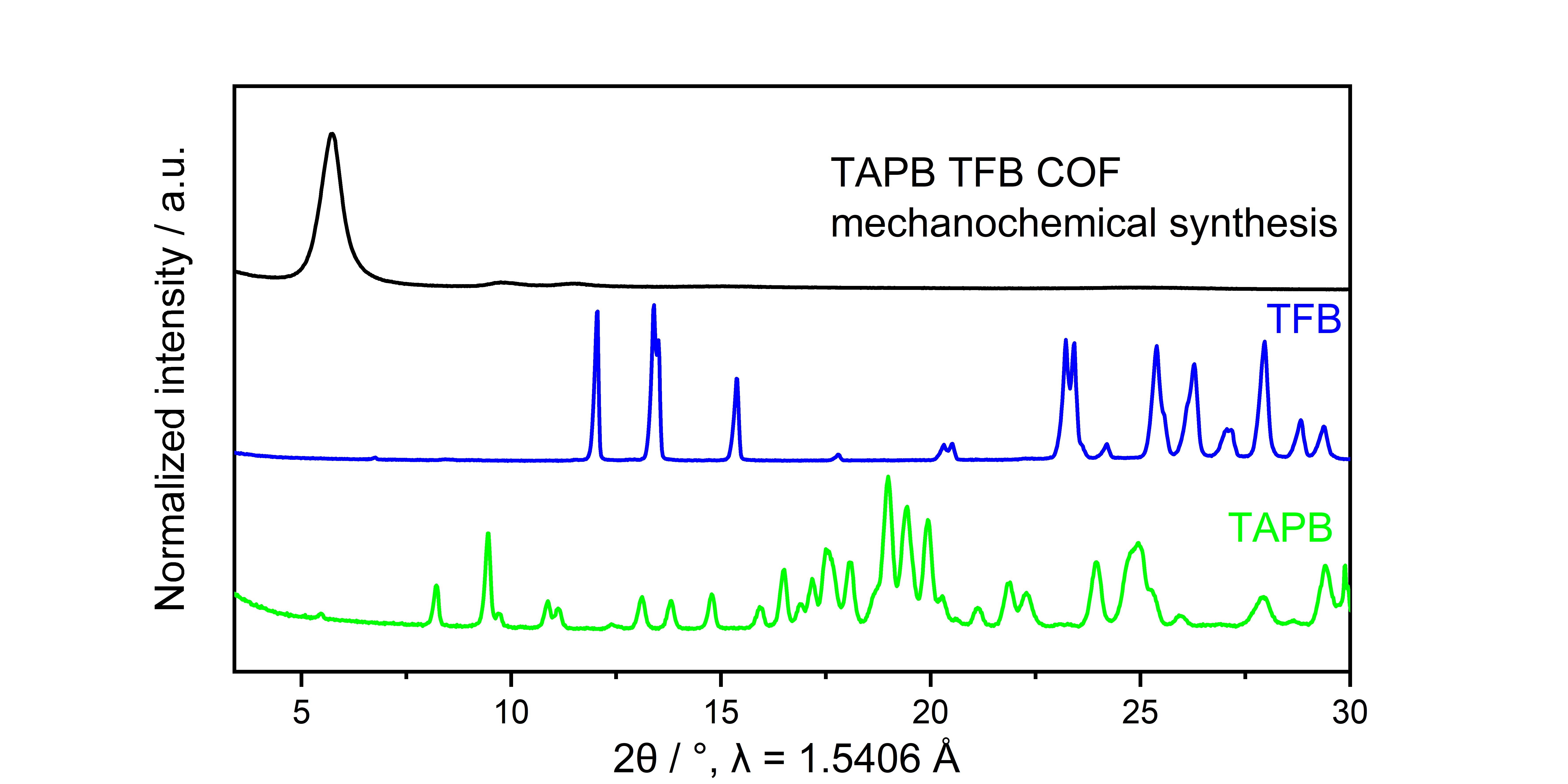
**

**Figure S1**: Comparison of XRD patterns for TAPB, TFB, and TAPB-TFB COF. The XRD of TAPB is shown in green, TFB in blue, and the product COF in black. The disappearance of the reactant peaks and the development of a new low-angle peak for the COF confirm the successful formation of TAPB-TFB COF.


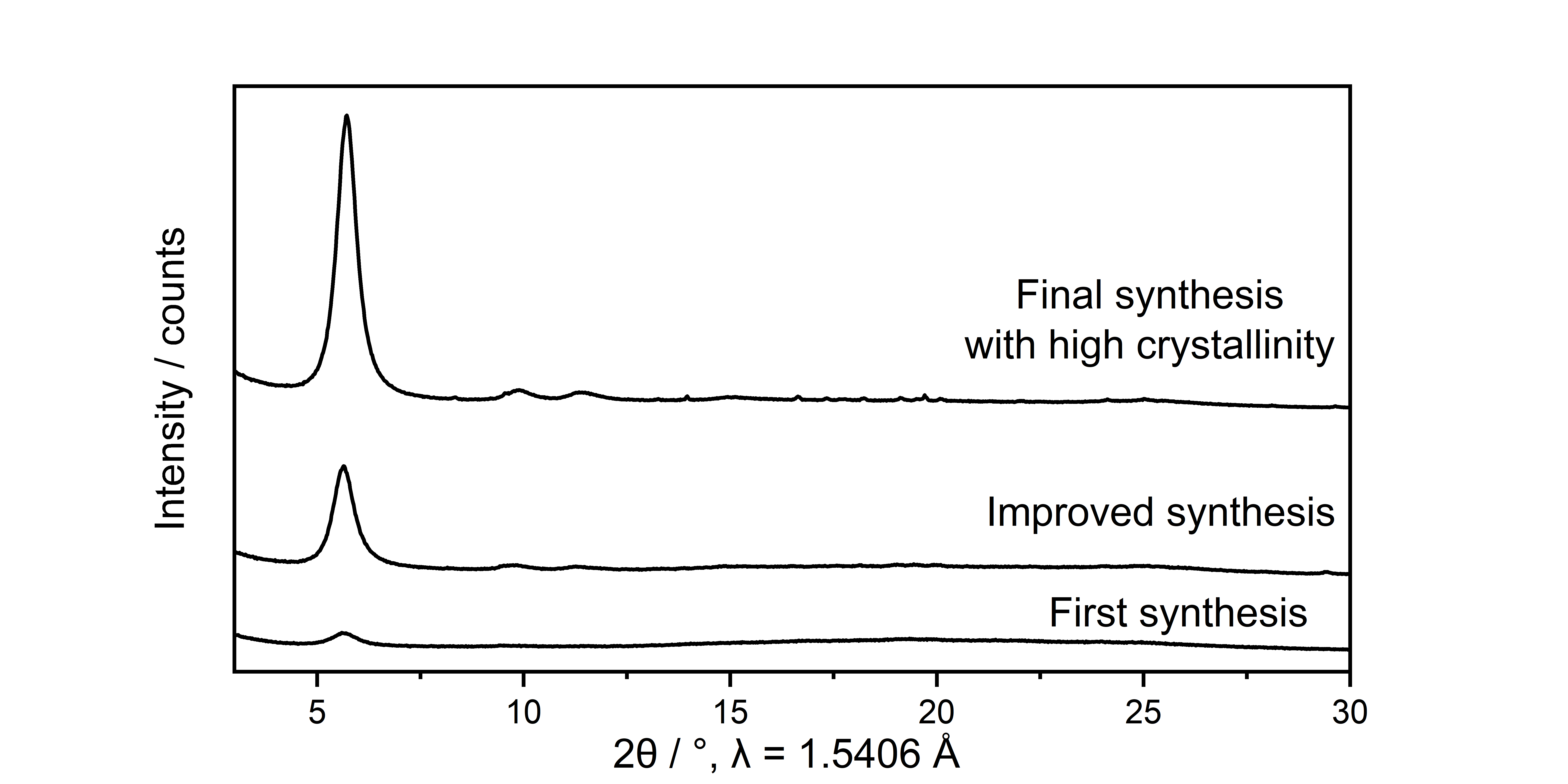


**Figure S2**: Comparison of XRD patterns highlighting the evolution of TAPB-TFB COF crystallinity across three stages: the first successful synthesis, the second-stage synthesis scaled up threefold with improved crystallinity, and the final optimized synthesis exhibiting the highest crystallinity. The optimal conditions for achieving maximum crystallinity were determined through systematic investigation of reaction parameters and real-time monitoring of COF formation at the DESY synchrotron.

***S4.2 In situ XRD***

***
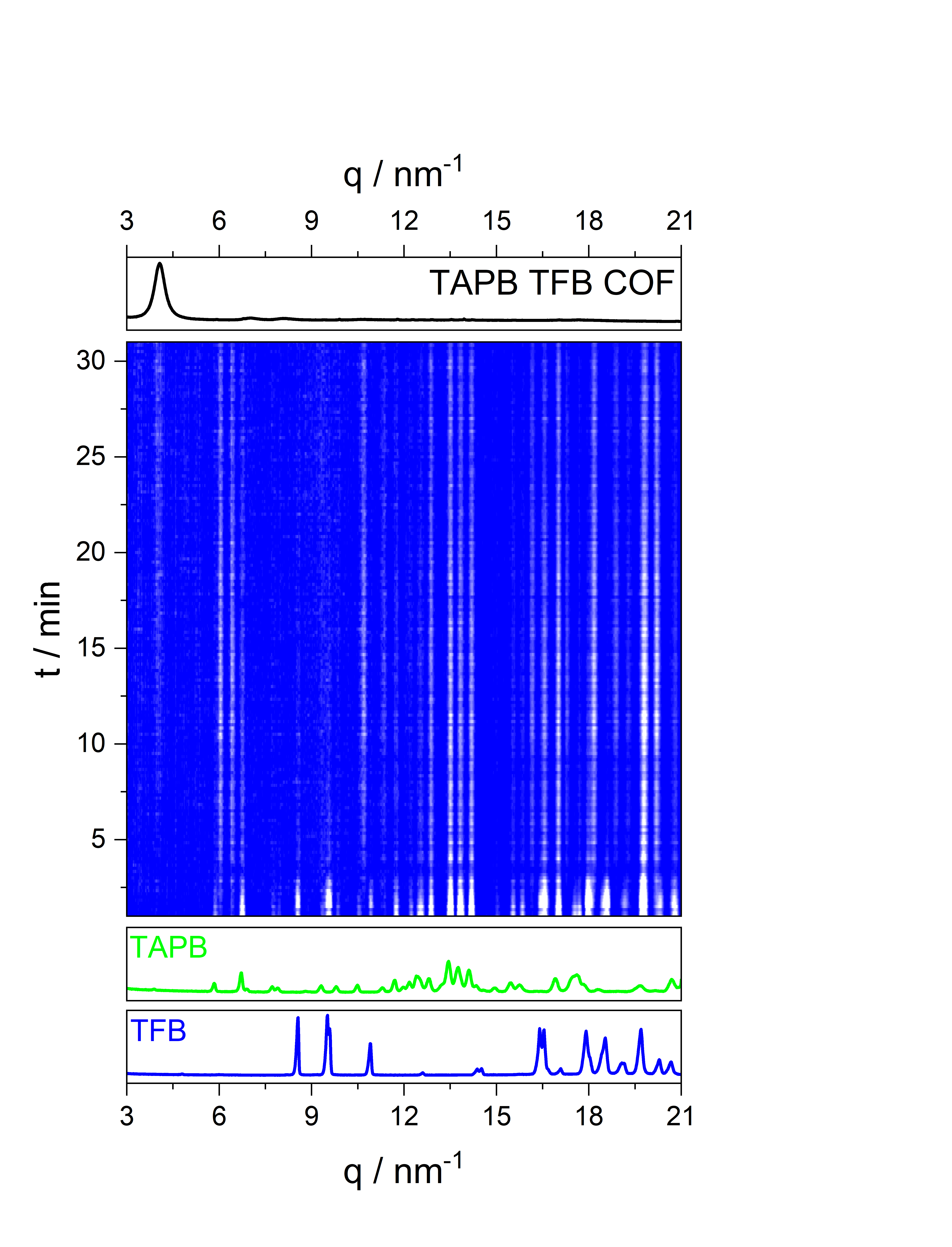
***

**Figure S3**: Time-resolved X-ray diffraction was employed to monitor the imine condensation reaction between TAPB and TFB, leading to the formation of TAPB-TFB COF under mechanochemical conditions. The reaction was conducted by milling 0.5 mmol of each reactant in a transparent 14 mL polymethyl methacrylate (PMMA) jar, with 112.5 µL of mesitylene and 150 µL of 6 M acetic acid as additives. Two 5 mm stainless steel balls were used as the grinding medium, operating at a frequency of 25 Hz. Notably, in contrast to the reaction depicted in Figure 2b (Main text), the frequency was decreased from 30 Hz to 25 Hz.

***S4.3 Influence of impact***

Influence of milling time was investigated. Prolonging reaction time i.e. having prolonged mechanical impact can lead to destruction of the framework resulting in lower crystallinity.


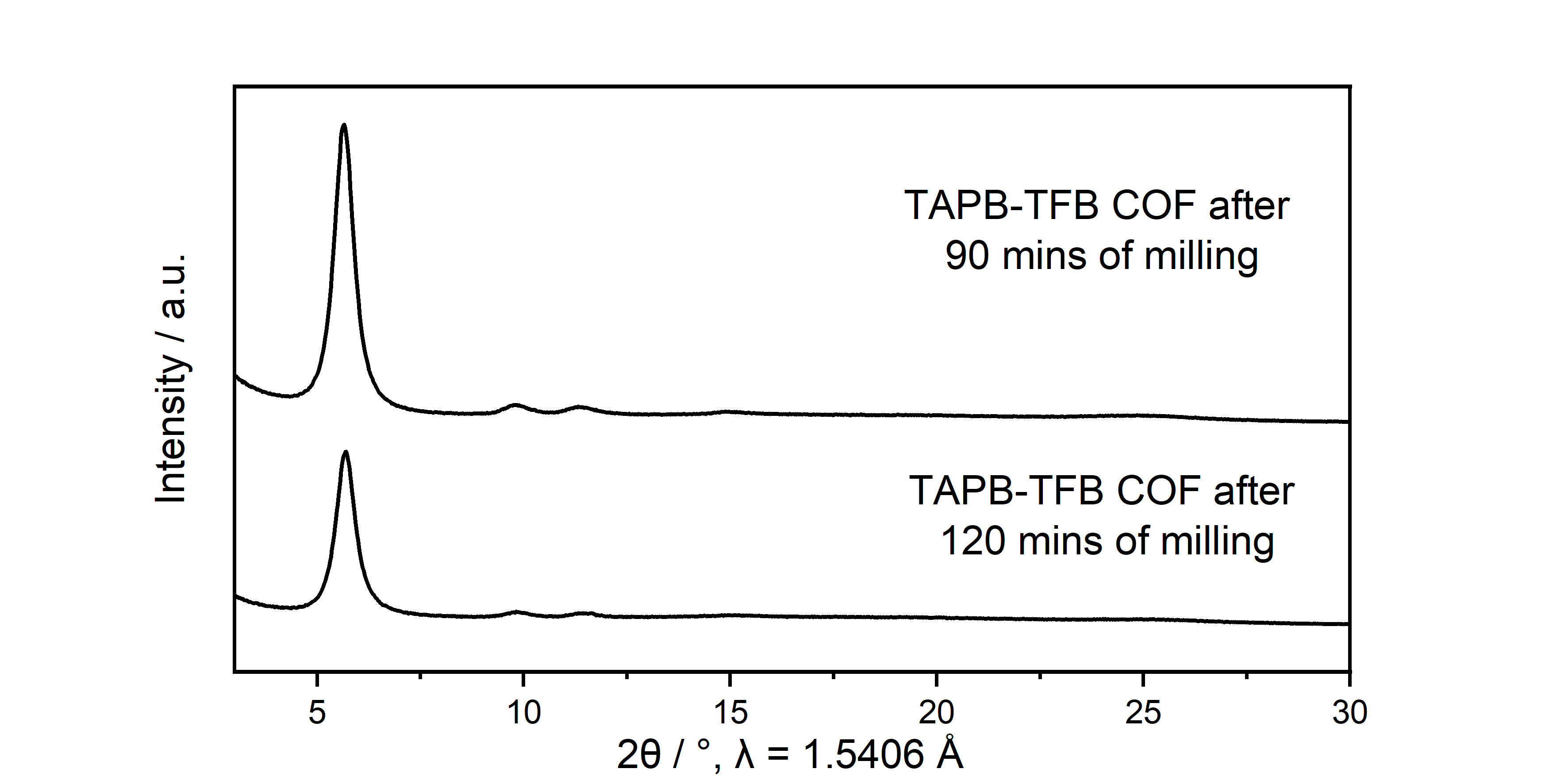


**Figure S4.** Comparison of XRD patterns for TAPB-TFB COF synthesized via mechanochemical for 120 minutes and 90 minutes.

Influence of the number of balls used in the mechanochemical synthesis was investigated. Reactions were carried out under standard conditions using different numbers of balls in a 10 mL jar (3, 4, 5, and 6 balls). XRD analysis showed that the reaction using 2 balls, the original standard condition, produced the best results, with no detectable peaks from unreacted starting materials. In all other cases, starting materials peaks were clearly visible. This suggests that while increasing the number of balls may enhance impact energy, it likely reduces mixing efficiency within the limited reaction volume. For this specific setup, using 2 balls appears to provide the optimal balance between sufficient mechanical energy and effective mixing to facilitate the reaction.


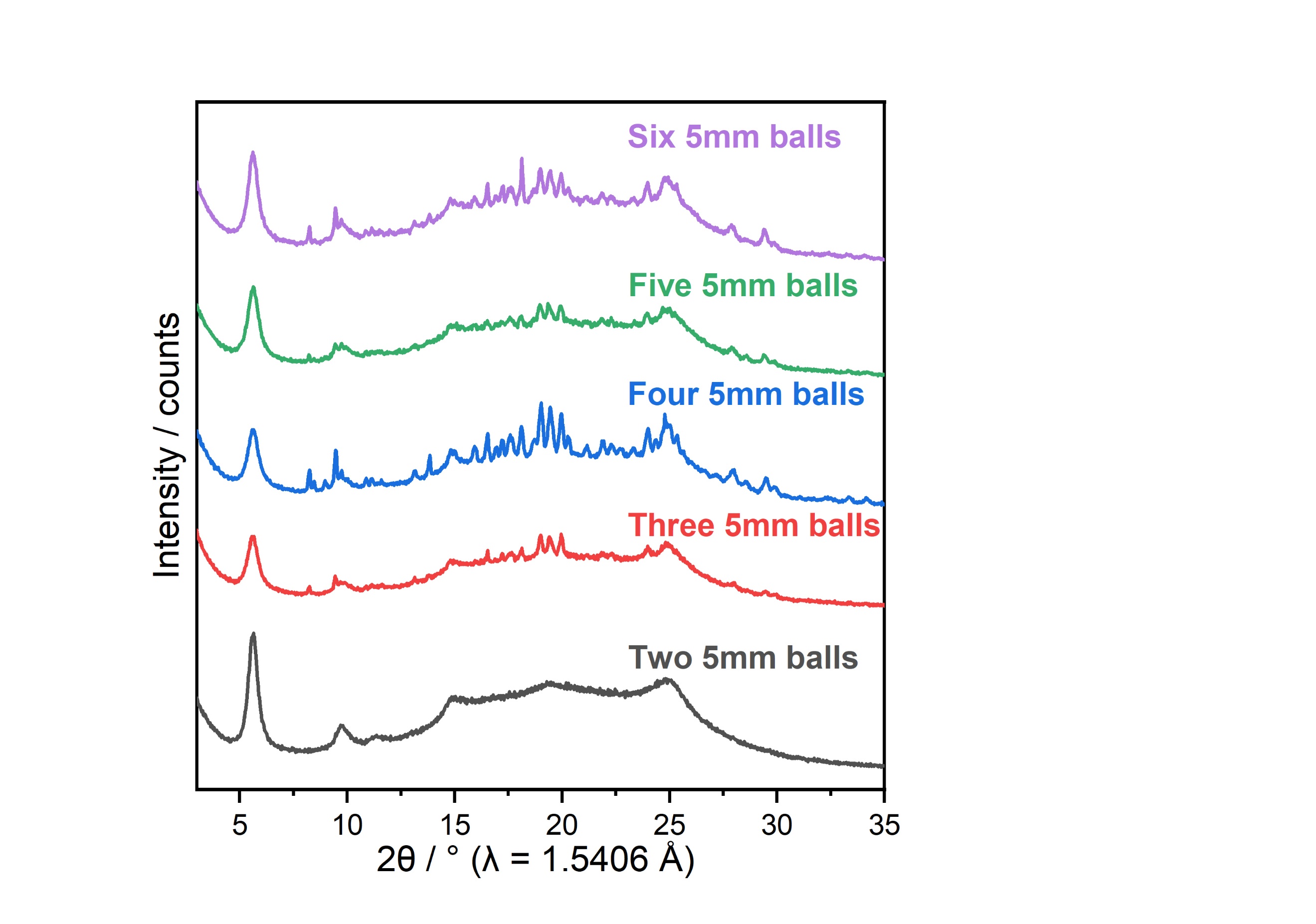

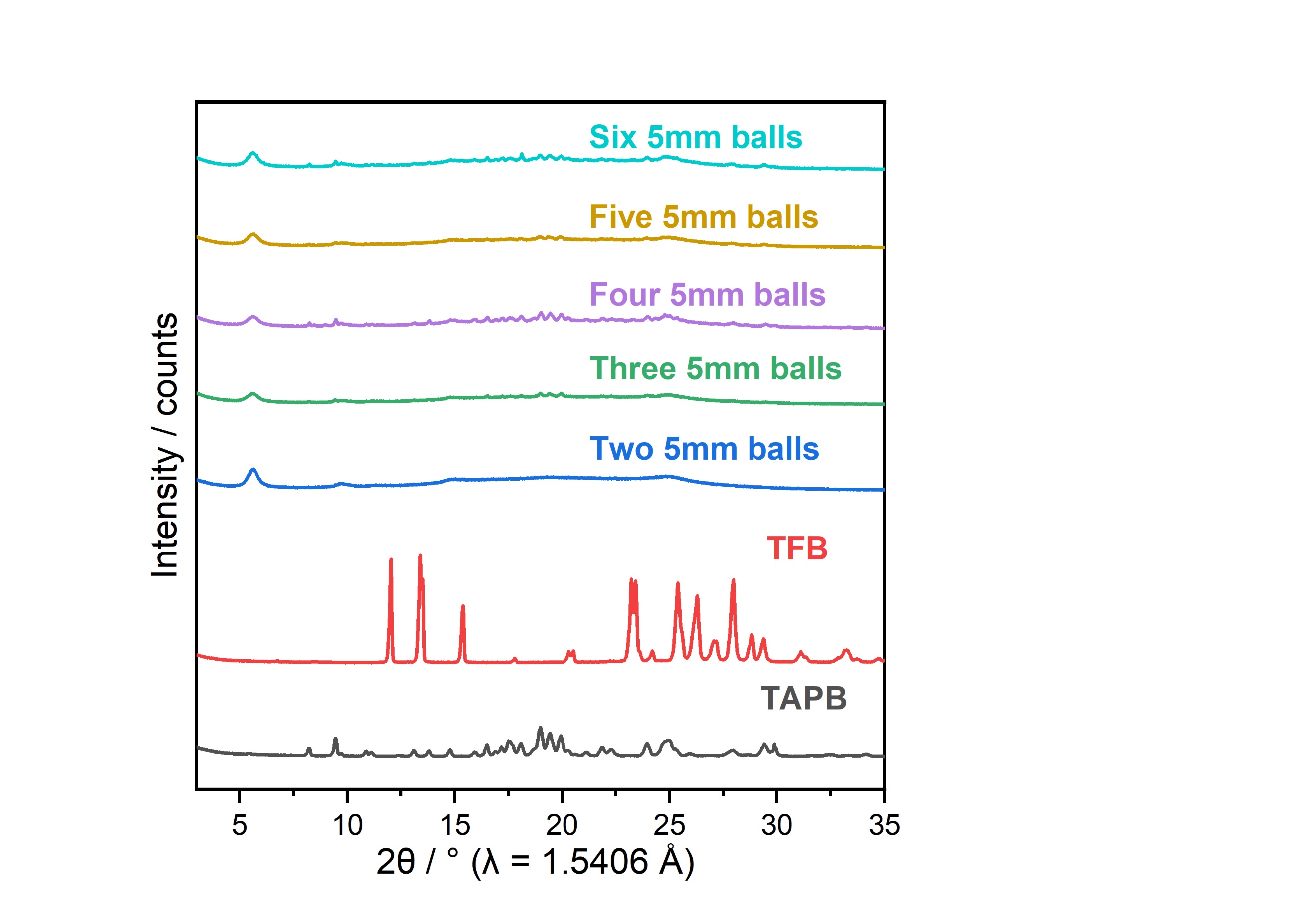


**Figure S5:** Left: XRD patterns comparing the starting materials, Tris(4-aminophenyl)benzene (TAPB) and Triformylbenzene (TFB) with COFs synthesized under standard reaction conditions (0.5 mmol reactants, 112.5 µL mesitylene, 150 µL 6 M acetic acid, 10 mL jar, two 5mm balls, 30 Hz, 90 min) using different numbers of 5 mm milling balls (3, 4, 5, and 6 balls). Right: Zoomed in view highlighting variations in crystallinity among COFs synthesized with different ball counts.

**S4.4 Effect of water and acetic acid**

A series of control experiments to assess its impact on the crystallinity of the COF was performed. Since water is generated as a byproduct during the reaction, we aimed to determine whether the intentional addition of water would influence the reaction outcome. Reactions were performed under our standard conditions (using mesitylene, 1,4-dioxane and acetic acid), supplementing the mixture with additional water in varying amounts (50 µL, 100 µL, and 150 µL). To isolate the effect of water as a solvent, reactions using only water as liquid additive were also carried out (100 µL, 200 µL, and 300 µL).

XRD analysis revealed that the standard reaction conditions yielded the highest crystallinity. In contrast, the reactions with added water showed diminished COF peak intensity and the presence of unreacted starting materials, with the latter becoming more pronounced as water content increased. In reactions where only water was used, no COF reflections were observed, and similarly, an increase in reagent signal intensity was seen with greater water volumes. All samples exhibited an amorphous hump, which we attribute to the lack of washing steps.


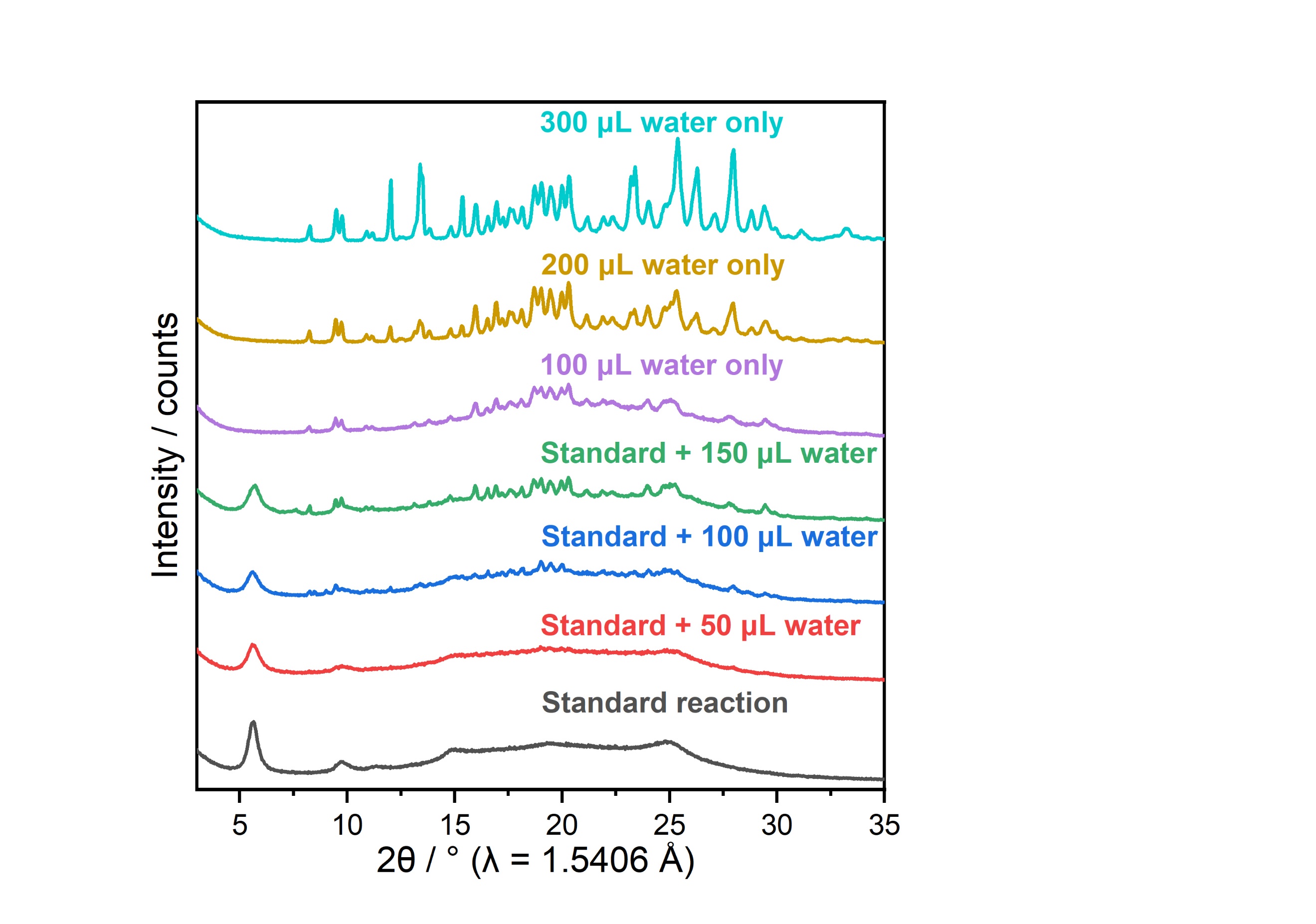

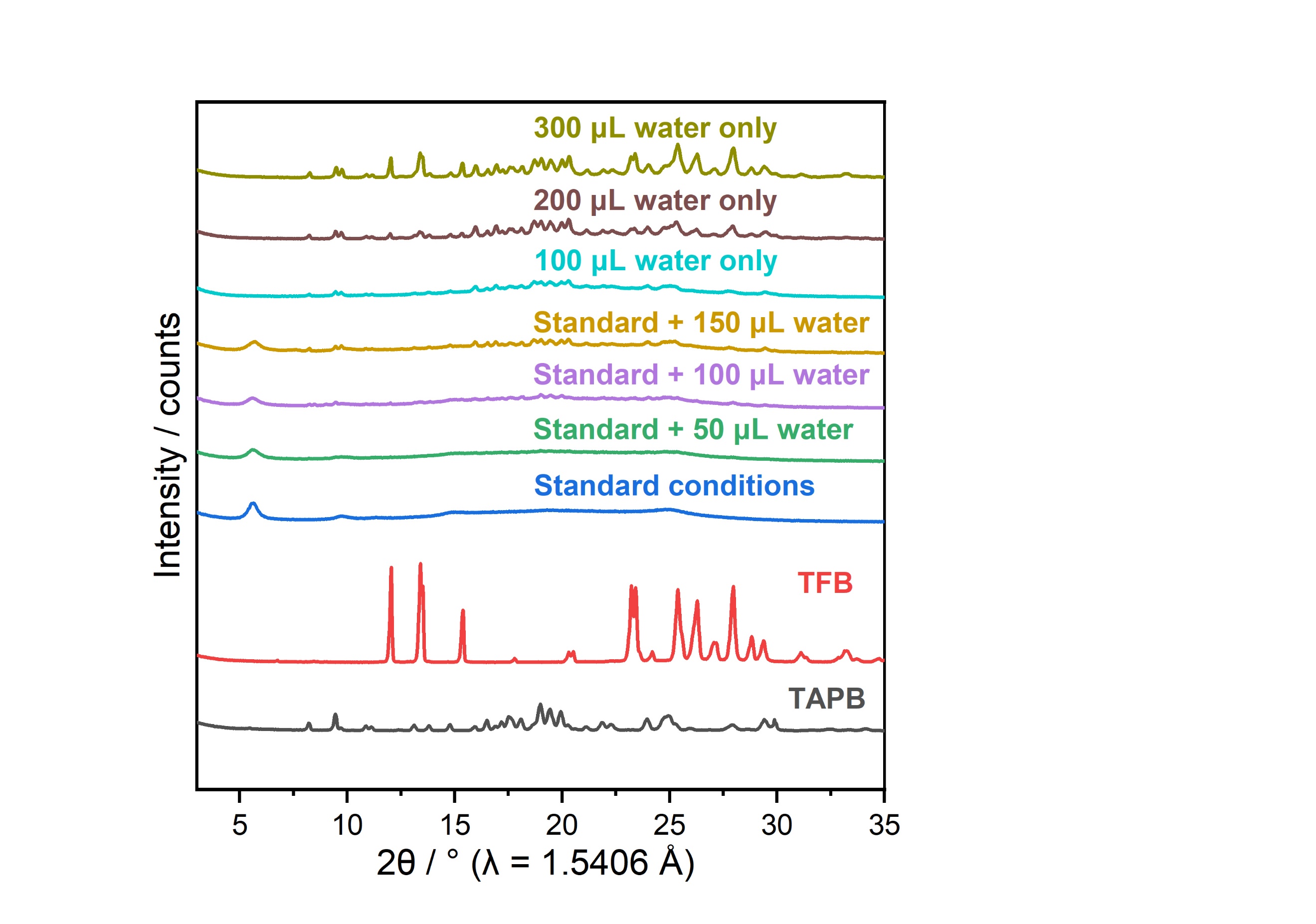


**Figure S6:** Left: Comparison of XRD patterns of the starting materials, 1,3,5-Tris(4-aminophenyl)benzene (TAPB) and 1,3,5-Triformylbenzene (TFB) with COFs synthesized under various conditions: standard conditions (0.5 mmol reactants, 112.5 µL mesitylene, 150 µL 6 M acetic acid, 10 mL jar, two 5 mm balls, 30 Hz, 90 min), standard + 50 µL water, standard + 100 µL water, standard + 150 µL water, and reactions using only water instead of mesitylene and 6M acetic acid (100 µL, 200 µL, and 300 µL). Right: Zoomed in view of the XRD patterns focusing on differences among the COFs synthesized under these varying conditions.

To investigate the role of acetic acid in the reaction, we conducted two additional experiments: one using 6 M acetic acid alone as the liquid additive, and another using a mixture of acetic acid and water, while keeping all other reaction conditions constant. In the case of acetic acid alone, XRD analysis showed significant amorphization of the starting materials, with only a very weak reflection at 2θ ≈ 5.6°, indicating that COF formation was minimal and that these conditions are insufficient for promoting proper framework growth. For the acetic acid and water mixture, we observed the formation of a dense agglomerated mass, commonly referred to as the “snowball effect”,^[2]^ which reduces mixing efficiency and mechanical impact. This was reflected in the XRD pattern, which showed the presence of unreacted starting materials and no evidence of COF formation. These results highlight the importance of carefully tuning the liquid additives to ensure both sufficient reactivity and effective mixing during mechanochemical synthesis.


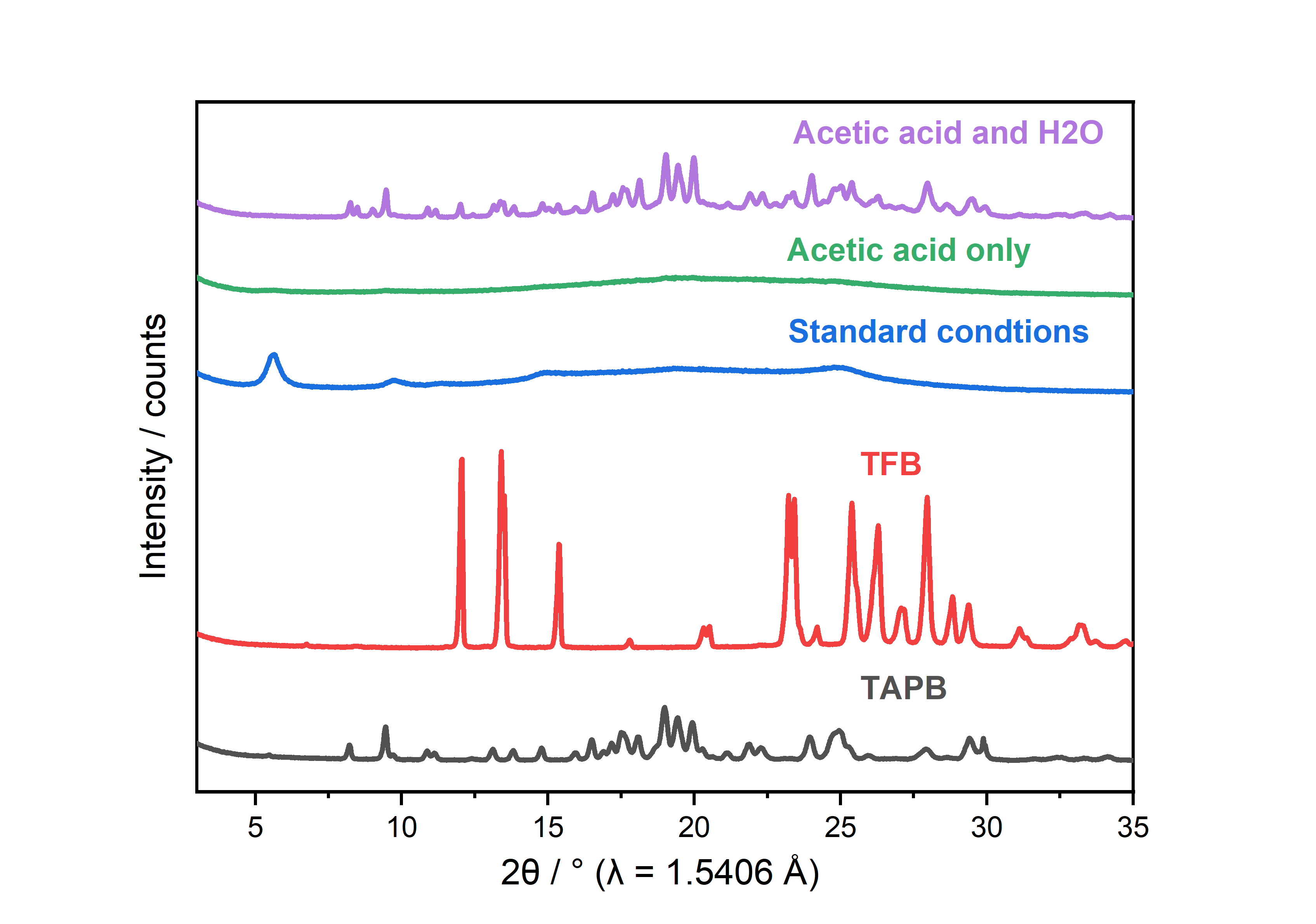


**Figure S7:** Left: Comparison of XRD patterns of the starting materials, 1,3,5-Tris(4-aminophenyl)benzene (TAPB) and 1,3,5-Triformylbenzene (TFB) with COFs synthesized under various conditions: standard conditions (0.5 mmol reactants, 112.5 µL mesitylene, 150 µL 6 M acetic acid, 10 mL jar, two 5 mm balls, 30 Hz, 90 min), using 150 µL acetic acid only and using 150 µL acetic acid and 150 µL water.

***S4.5 Mechanochemical and solvothermal COF***

**
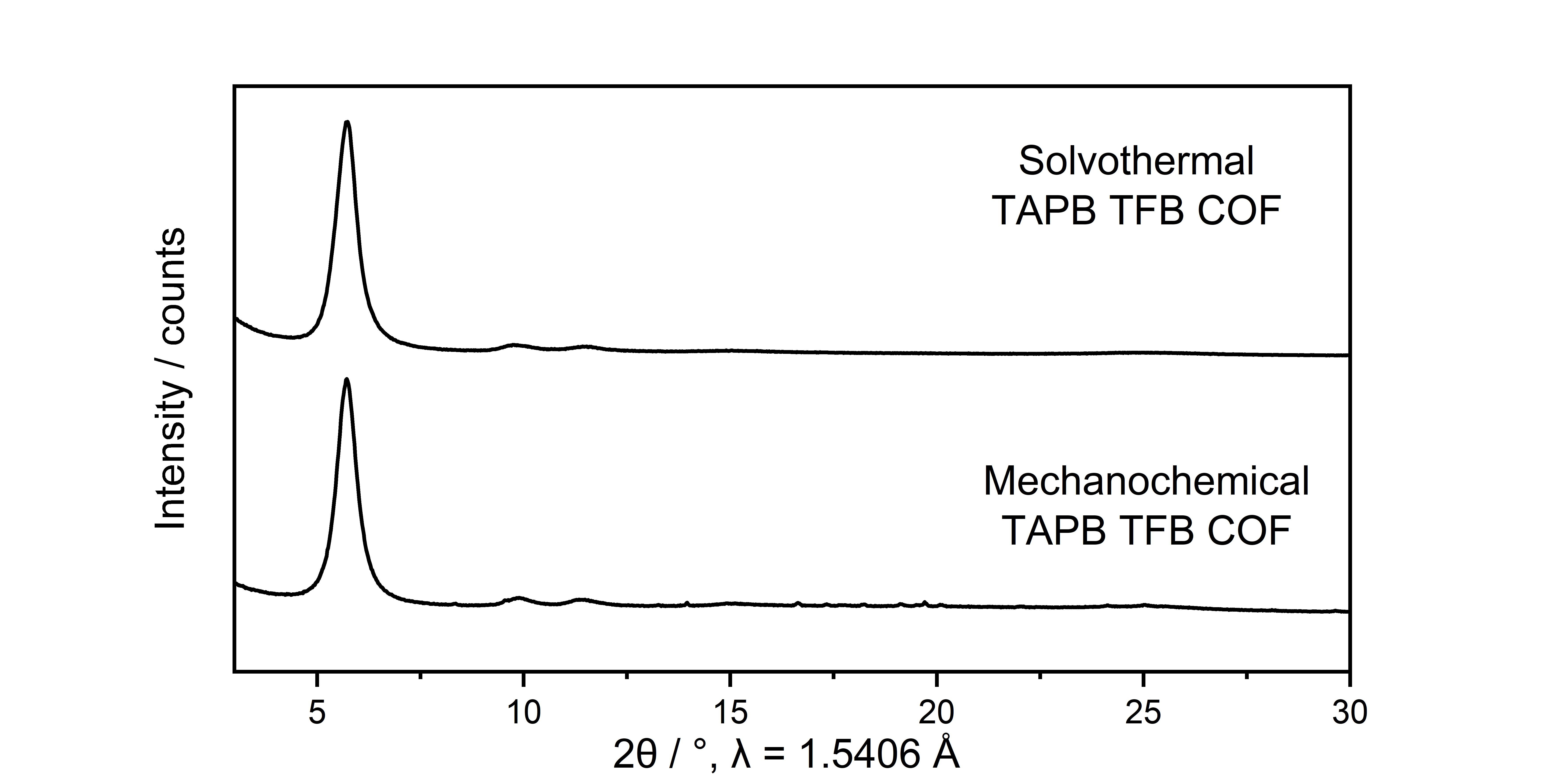
**

**Figure S8:** Comparison of XRD patterns for TAPB-TFB COF synthesized via mechanochemical and solvothermal methods. Both methods yield reflections at the same 2θ angles, indicating comparable structural frameworks and order.

***S4.6 Simulated structure***

Materials Studio is a modelling and simulation software employed to simulate materials at the atomic and molecular levels. The software offers an extensive suite of tools for visualising, constructing and analysing material structures, thereby facilitating the prediction of properties and optimisation of designs. The software's user-friendly interface and advanced simulation capabilities enabled me to gain valuable insights into the behaviour and characteristics of TAPB-TFB COF.


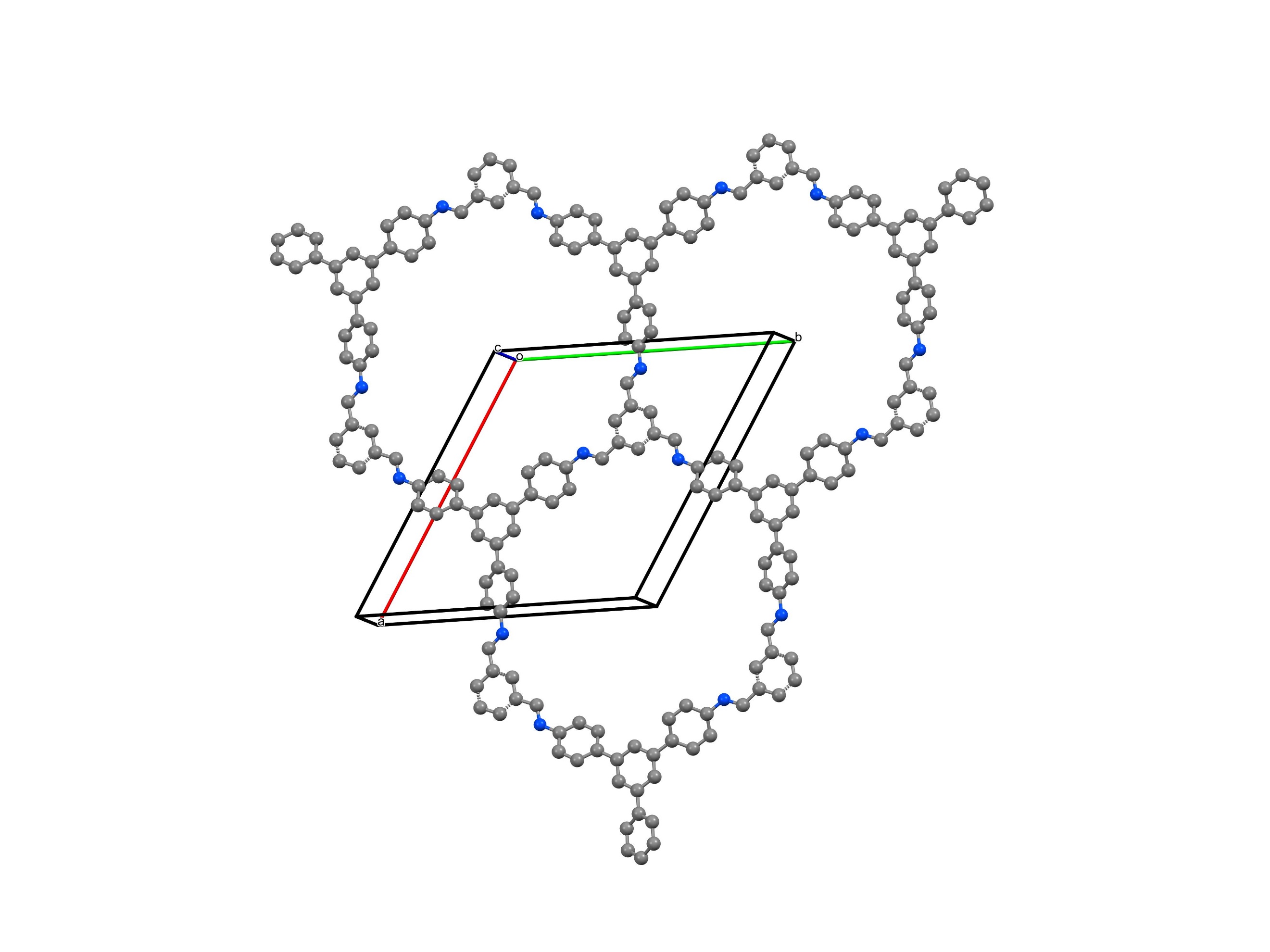


**Figure S9**: Simulated structure of TAPB TFB COF generated using Materials Studio, modeled in the trigonal P3 (143) space group. The unit cell parameters are as follows: a = b = 18.0241 Å, c = 3.5615 Å, α = β = 90 °, γ = 120 °), and a volume of 1002.0070 Å^3^.

***S4.7 Nitrogen adsorption***

Nitrogen adsorption measurements were employed to determine the BET specific surface area of the material. The adsorption-desorption isotherm, presented in Figure 2, reveals the characteristic adsorption behavior. Using this data, the BET surface area was calculated to be 202.19 m²/g. Several key points were considered in this calculation, including the linearity of the BET plot, the selection of the appropriate pressure range, and the assumption of monolayer adsorption on the surface. These factors ensured accurate determination of the specific surface area, providing insight into the material's porosity and surface characteristics. The following points were considered for the calculation of BET surface area:

**Table 1**: Key parameters for the calculation of the BET specific surface area of the material, determined through nitrogen adsorption measurements.

| **Relative Pressure (p/p°)** | **1/[Q(p°/p - 1)]** |
| --- | --- |
| 0,00761 | 1,99765E-4 |
| 0,01271 | 3,17801E-4 |
| 0,01837 | 4,42552E-4 |
| 0,0242 | 5,64311E-4 |
| 0,03084 | 7,03778E-4 |
| 0,03839 | 8,66339E-4 |
| 0,04615 | 0,00103 |
| 0,05401 | 0,0012 |
| 0,06222 | 0,00138 |


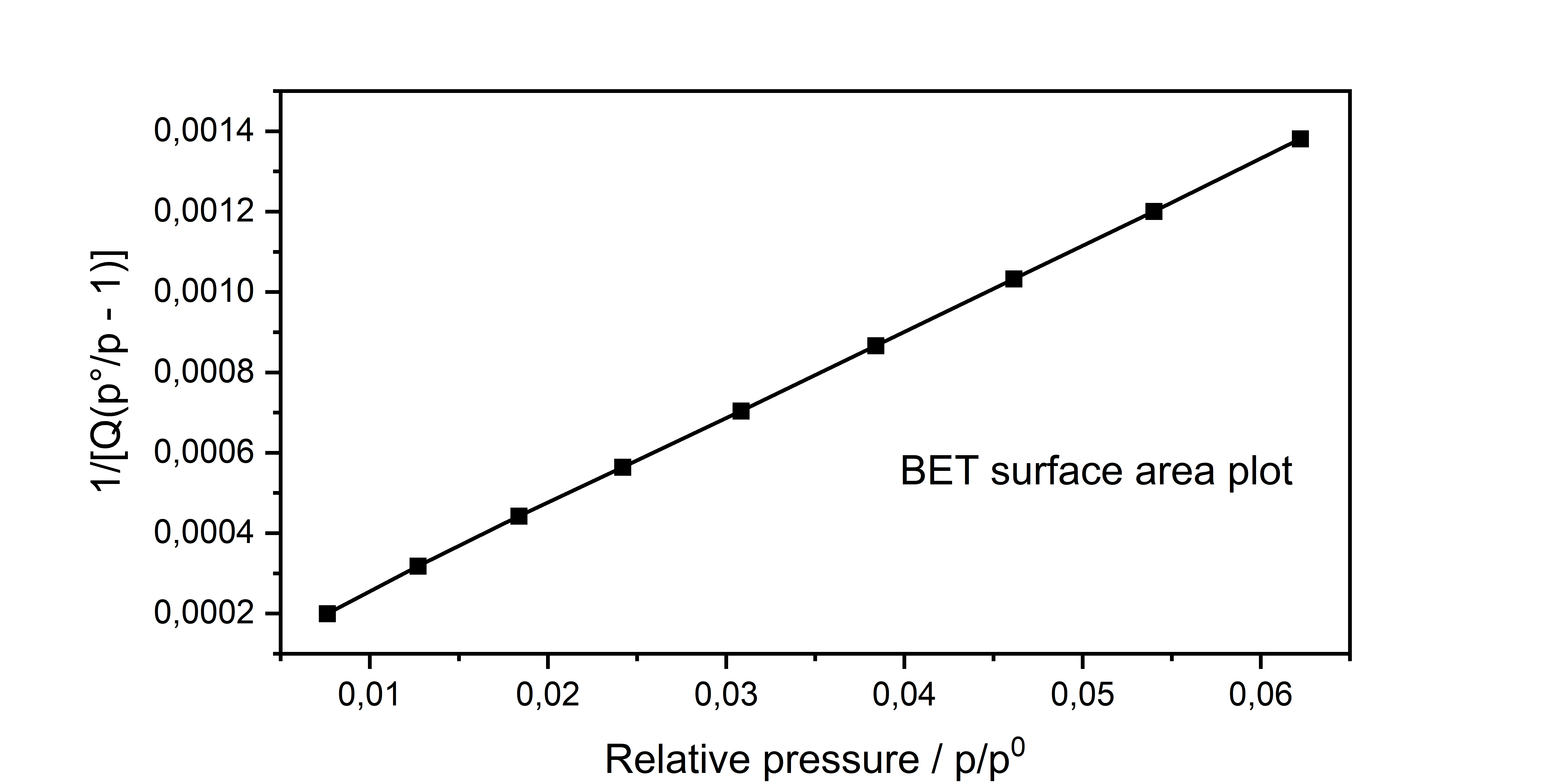


**Figure S10**: BET specific surface area plot for TAPB TFB COF. The calculated BET surface area is 202.19 m²/g, derived from the analysis of the isotherm. Key considerations for this calculation included the linearity of the BET plot, the selection of the appropriate pressure range, and the assumption of monolayer adsorption.

The following Rouquerol plot was plotted using the following points:

**Table 2**: Key parameters for determining the Rouquerol BET plot

| **Relative Pressure (p/p°)** | **Q (1 - p/p°) (cm³/g STP)** |
| --- | --- |
| 0,00761 | 38,1149 |
| 0,01271 | 39,9885 |
| 0,01837 | 41,5172 |
| 0,0242 | 42,8824 |
| 0,03084 | 43,8176 |
| 0,03839 | 44,3132 |
| 0,04615 | 44,6836 |
| 0,05401 | 44,9799 |
| 0,06222 | 45,0617 |


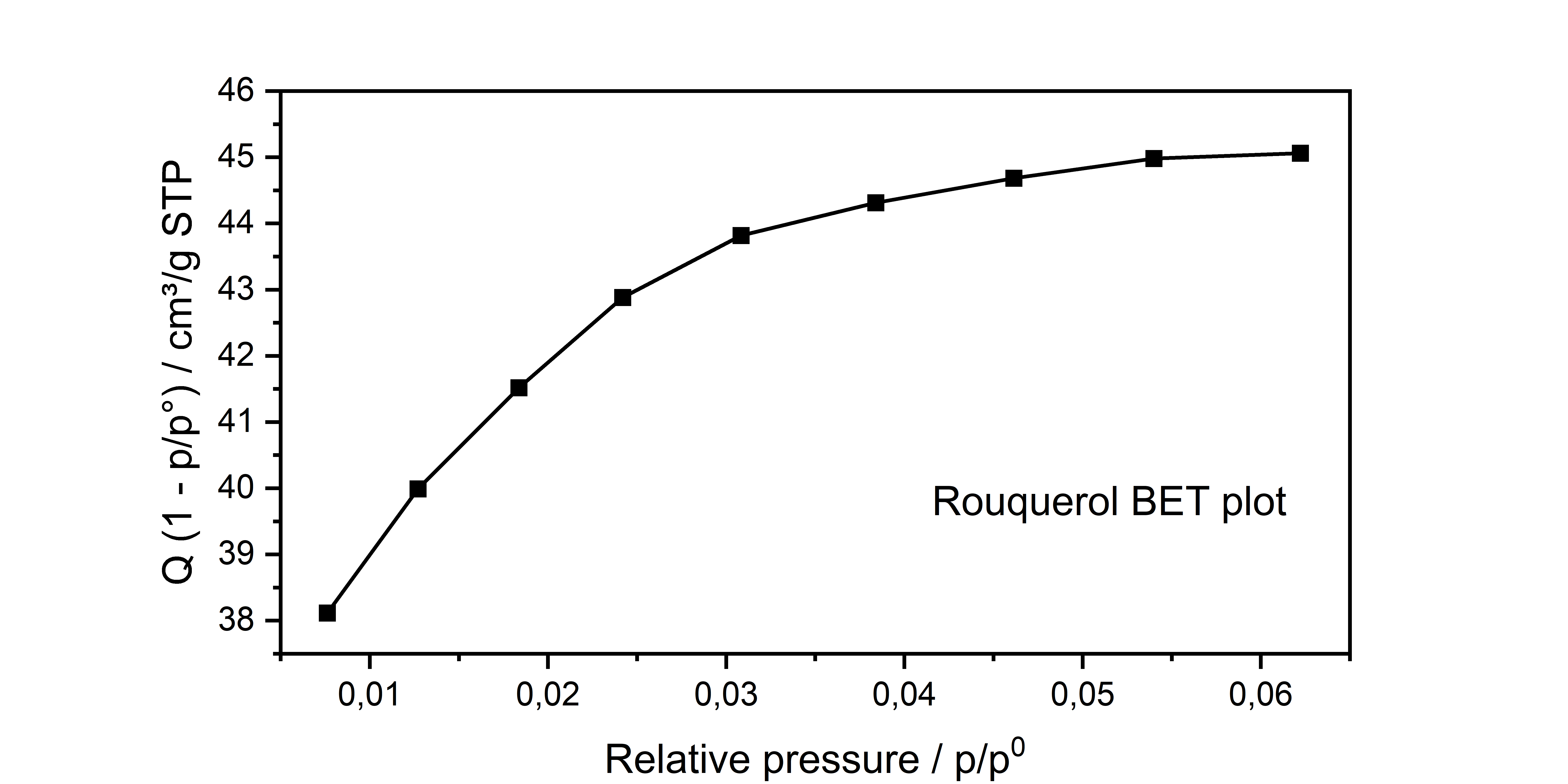


**Figure S11**: Rouquerol BET plot.

The pore width distribution was determined using the Horvath-Kawazoe method, based on the cumulative pore volume plot, with spherical pore geometry selected for the analysis. The maximum pore volume was calculated to be 0.0737 cm³/g at a relative pressure of 0.054. Furthermore, the median pore width was determined to be 1.254 nm. These values provide critical insights into the material’s microporosity and its capacity for gas adsorption, highlighting its potential for applications where precise control of pore size and volume is essential.

***S4.8 SEM***

SEM images reveal significant particle aggregation across different magnifications. In the first image, numerous small particles with no distinct or regular geometry are observed. Upon further magnification, the second and third images show particles in the range of several hundred nanometers aggregating into larger clusters. Despite this increase in particle size, no specific geometric patterns are discernible, suggesting a highly irregular aggregation process. This lack of uniformity is likely attributed to the mechanochemical milling process, which introduces mechanical forces that break down the material into ultrafine particles. However, these particles exhibit a strong tendency to reassemble into larger aggregates, potentially due to inter-particle forces such as van der Waals interactions or residual stresses. The milling, while effective at reducing particle size, appears to compromise the retention of any distinct geometric features, resulting in the formation of irregular, loosely bound aggregates.

**
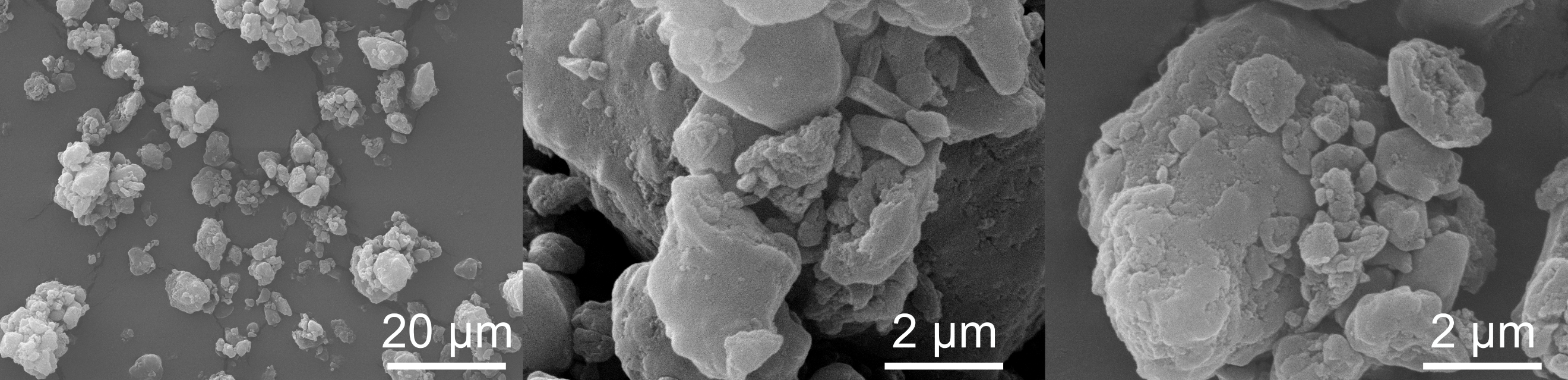
**

**Figure S12**: SEM images showing particle aggregation at different magnifications. The first image reveals small, irregular particles, while the second and third show nanometer-sized particles clustering into larger aggregates. No distinct geometry is observed, likely due to mechanochemical milling, which reduces particle size but leads to irregular, loosely bound aggregates.

***S4.9 TEM***

Transmission electron microscopy (TEM) successfully isolated individual flat particles, a notable contrast to the aggregation observed in SEM imaging. This improved isolation is attributed to the specific sample preparation method used for TEM analysis. A small amount of powder was further milled using mortar and pestle and suspended in deionized water, followed by ultrasonication to break up particle agglomerates and promote uniform dispersion. A drop of the well-dispersed suspension was then deposited onto a TEM copper grid and allowed to dry, facilitating the visualization of isolated particles. Despite these preparative measures, large aggregates were still observed in some regions, although individual flat particles were also distinguishable. These flat particles appeared crumpled, resembling folded layers of covalent organic framework (COF) material.


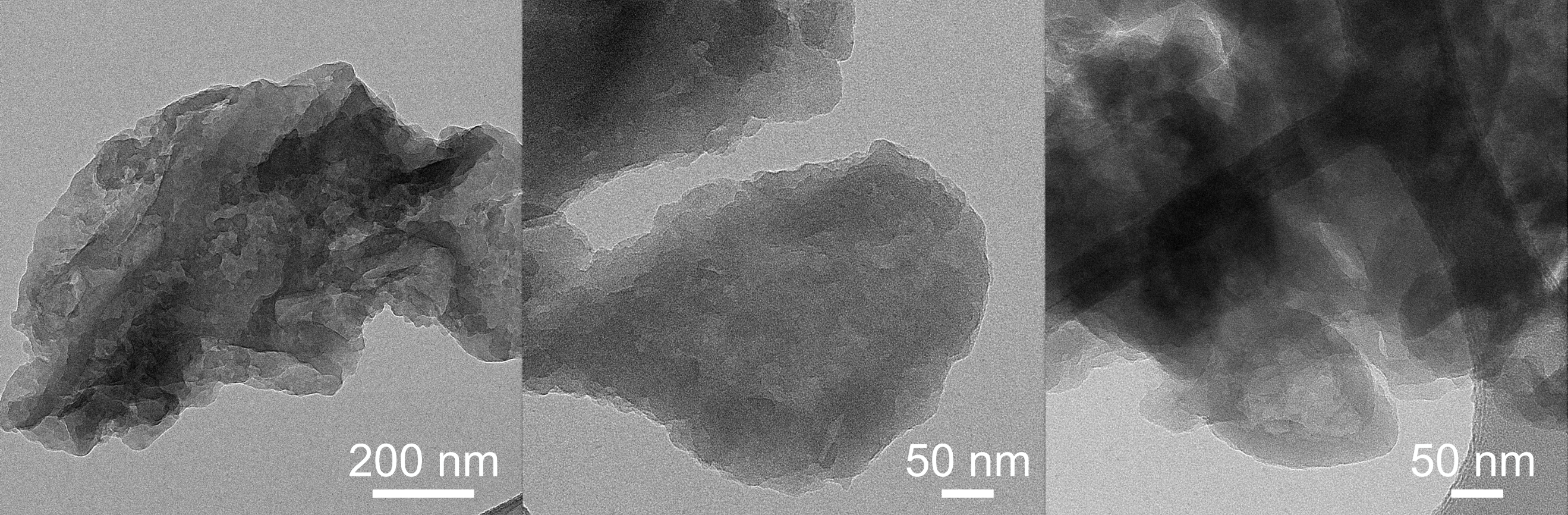


**Figure S13**: TEM images demonstrating the successful isolation of individual flat particles, achieved through careful sample preparation. The powder was milled, suspended in deionized water, and ultrasonicated to enhance dispersion. The images reveal crumpled layers of covalent organic framework (COF) material alongside larger aggregates.

**S5. Stability test**

Before conducting adsorption tests, the stability of the TAPB-TFB COF in various media was evaluated. A total of 50 mg of COF was shaken in three different solutions: water, PFOA (50 µg/L), and PFOS (50 µg/L). This was done using an orbital shaker with 50 mL polypropylene centrifuge tubes, following the same conditions as the adsorption experiments. Each tube contained 30 mL of solution, and the COF was shaken at 400 rpm for 7 days. After shaking, the COF was collected using filter papers and analyzed by XRD. The resulting XRD patterns showed intense peaks, confirming that the COF retained both its structural integrity and crystallinity, demonstrating its stability in these media.

**
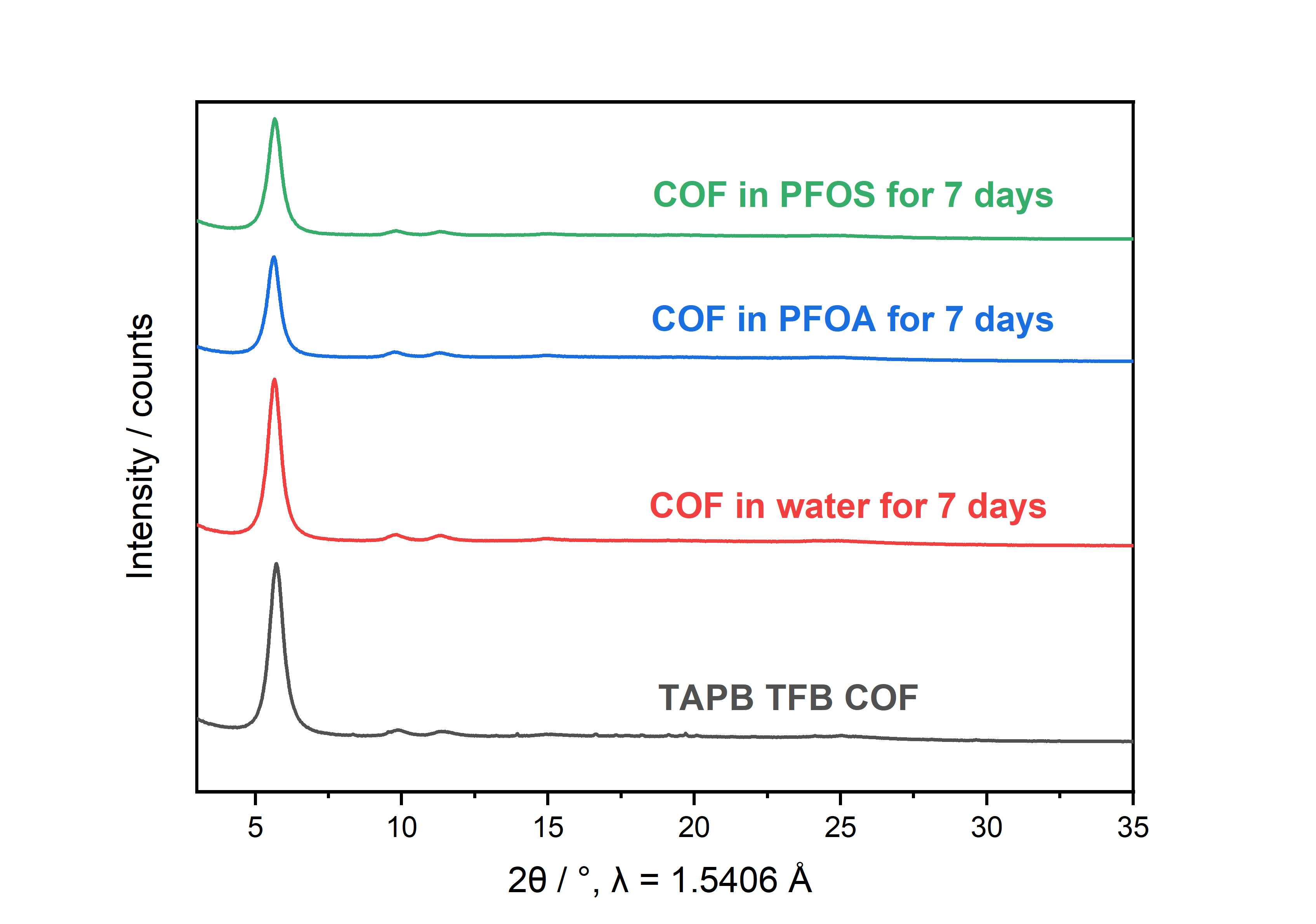
**

**Figure S14**: XRD analysis of TAPB-TFB COF after stability testing in water, PFOA, and PFOS solutions (50 µg/L). The COF was shaken at 400 rpm for 7 days, and the XRD patterns show intense peaks, confirming that the COF retained its structure and crystallinity in all tested media, demonstrating its stability.

Further experiments were conducted to assess the chemical stability of the TAPB-TFB COF under a range of pH conditions. The COF was exposed to aqueous solutions at pH 1, 2, 4, 10, 12, and 14, and the resulting materials were analyzed by XRD. A general reduction in peak intensity was observed in all cases compared to the pristine COF, which is expected due to partial hydrolysis of the imine bonds under extreme pH conditions. However, it is noteworthy that the COF retained significant crystallinity across the entire range, except for pH 1, where structural degradation was more pronounced.

These results demonstrate that the TAPB-TFB COF exhibits good stability under both fairly acidic and highly alkaline conditions. Given that typical water treatment environments fall within a near-neutral pH range, the COF remains structurally stable under relevant application conditions.


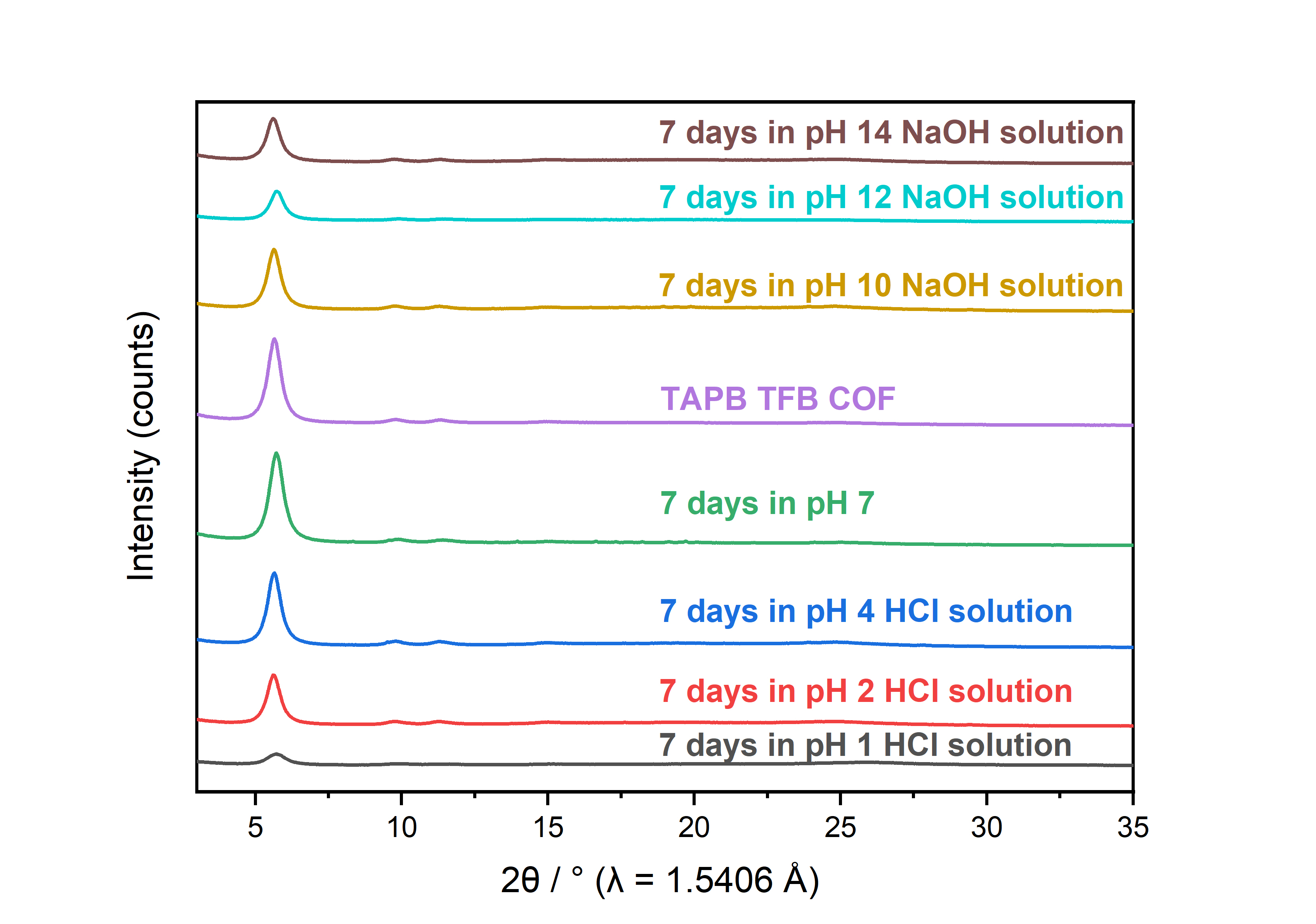


**Figure S15:** XRD patterns of the pristine COF compared with samples exposed for 7 days to aqueous solutions of varying pH (1, 2, 4, 7, 10, 12, and 14). The COF retains its characteristic crystalline peaks across all conditions, with the exception of pH 1, where a significant reduction in peak intensity is observed, indicating partial structural degradation.

**S6 Sulphur 1s XPS of PFOS adsorbed COF**


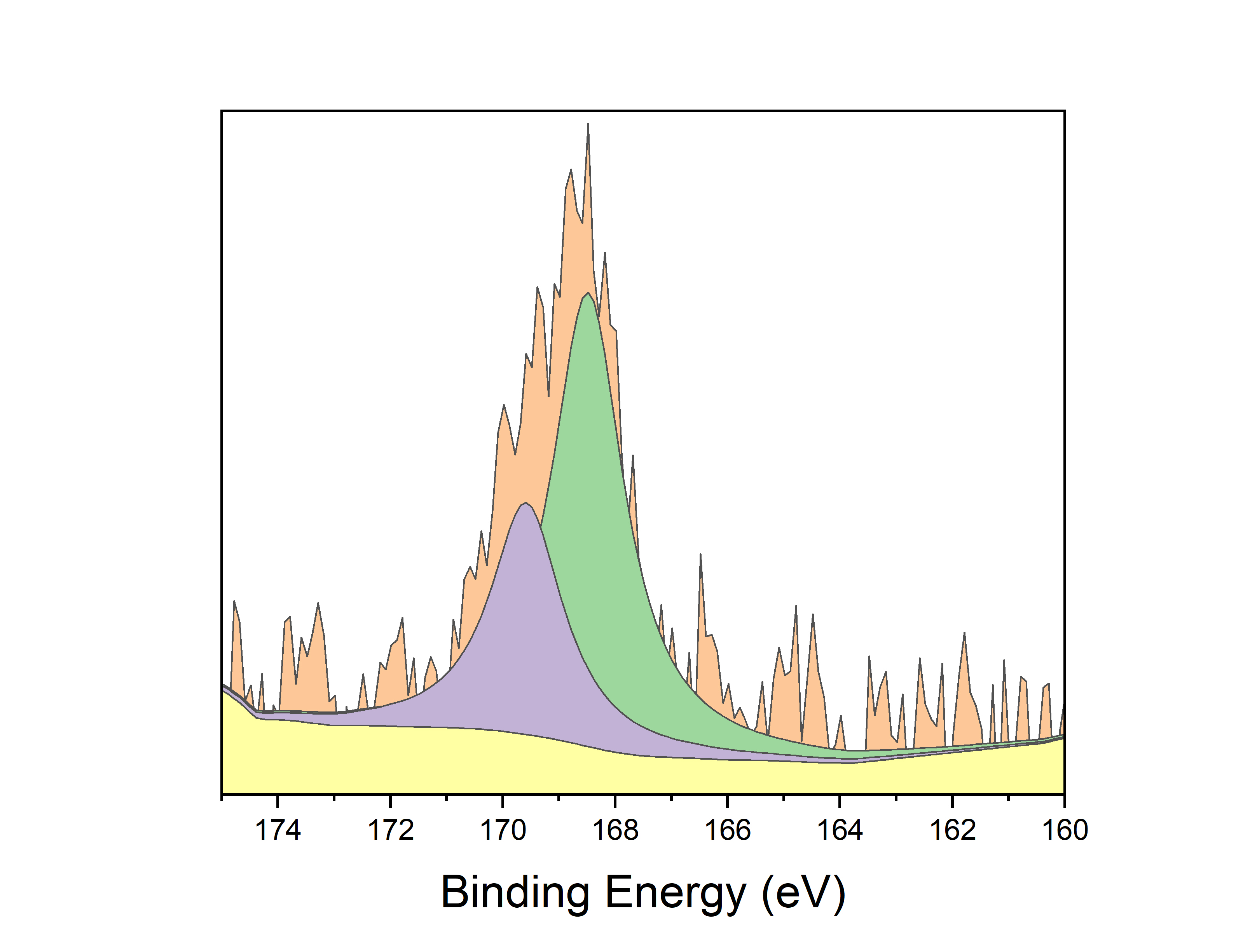


**Figure S16:** Sulphur 1s XPS spectrum showing two peaks, one at 168.5 eV, attributed to sulfonate groups, and another at 169.6 eV, indicative of oxidized sulfur species.

**S7 DFT and MLIP Simulations**

As described in the main text, we performed additional density functional theory (DFT) and machine-learned interatomic potential (MLIP) simulations.

To construct the simulation models, the COF structure was duplicated in the z direction (i.e., in the direction of the layer stacking). Then, one PFOA molecule with a deprotonated carboxylic acid group was inserted and placed close to one of the COF's protonated imine groups In both cases, the positions of the PFOA molecule plus the additional proton on the imine were fully optimized while the positions of the COF and the lattice parameters were fixed to the values generated by Materials Studio. This was done at the MACE-MPA-0 level^[3]^ (MLIP based on the MACE architecture trained on the Materials Project^[4,5]^ and Alexandria database^[6]^) and the DFT-PBE level, including the D3(BJ) dispersion correction in both cases. To compute the interaction energy, the energy of a system where the PFOA anion was moved to the middle of the pore. Then, we evaluated the energy difference. Additionally, we repeated the same procedure for a charge-neutral PFOA and the non-protonated imine. Here, we computed the interaction energy relative to an isolated COF framework and a fully optimized PFOA molecule (optimization performed in a 25 Å×25 Å×25 Å unit cell with vacuum around the PFOA). This amounts to an interaction energy of -110 kJ/mol at the DFT level. While this is likely not the hydrogen bond occurring in the actual adsorption process, it also confirms the imine group's ability to form strong hydrogen bonds.

In addition, we conducted steered molecular dynamics (SMD) simulations^[7]^ to gain further confidence that the interaction between the deprotonated carboxyl group of PFOA and the protonated imine group of COF-1 is indeed energetically favorable. In these simulations, the center of mass of the two oxygens of the carboxylic group is pulled towards the protonated nitrogen of the imine group along a virtual spring with constant velocity. The work (W) required to pull the head group can be related to the change in the free energy (ΔG) following Jarzinsky’s equality^[8]^: $\left\langle e^{-\beta W} \right\rangle=e^{-\beta\Delta G}$, where β=1kBT with kB being the Boltzmann constant and T the temperature. The work is calculated as: $W\left( x\left( t \right) \right)= \int_{0}^{x(t)} F\left( t \right)dx(t)$, where t is time, x(t) is the spring extension and F(t) is the pulling force defined as: $F\left( t \right)=2k (v*t-s\left( t \right))$. Here, k is the force constant (0.05 eV/Å²), v is the constant pulling velocity (-0.004 Å/ps), and s(t) is the distance between the current attachment point and the initial one. During the pulling process, the positions of the COF atoms are restrained using harmonic potentials to ensure accurate targeting of the nitrogen atom. We performed five pulling simulations, where each simulation lasted 1 ns with a time step of 1 fs. The temperature was maintained at 298 K using a Langevin thermostat with a damping constant of 100 fs. All simulations were carried out using the LAMMPS simulation package,^[9]^ adapted for use with MACE machine learning models. Specifically, we employed the large MACE-OFF23 model^[10]^ to describe interatomic interactions. MACE-OFF23 is a short-range potential designed to replicate the energy predictions of the ωB97M-D3(BJ)/def2-TZVPPD level of quantum mechanics.^[11–14]^ While it does not explicitly account for electrostatic interaction, it remains sufficiently accurate for qualitative analysis. As such, MACE-OFF23 can still provide valuable insights into the nature of favorable interactions between functional groups. The starting structure of COF-1 for the simulations has been generated by duplicating the protonated and optimized COF structure in x- and y- direction, in addition to the duplication in z-direction, described earlier. In Figure S17 we show the change in free energy as a function of decreasing spring length. The shortest spring length corresponds to proximity between the protonated nitrogen and the center of mass of the carboxylic group. As one sees, in the beginning the free energy decreases with decreasing spring length until it reaches a minimum of -0.11 eV (~-10.6 kJ/mol) at a distance of ~3.75 Å. This indicates that it is indeed energetically more favorable for the system if the two functional groups approach each other. However, if the distance gets too short, we observe an increase in the free energy again, indicating less favorable interactions at shorter distances. Thus, the SMD simulations qualitatively agree with the DFT calculations, where proximity between COF-1 and PFOA is energetically more favorable than having PFOA in the middle of the cell. However, the energy values of the two different modeling approaches cannot and should not be directly compared. They should rather be interpreted as complementary approaches, indicating that it is more favorable for the system to have PFOA close to the protonated imine group rather than having it in the center.


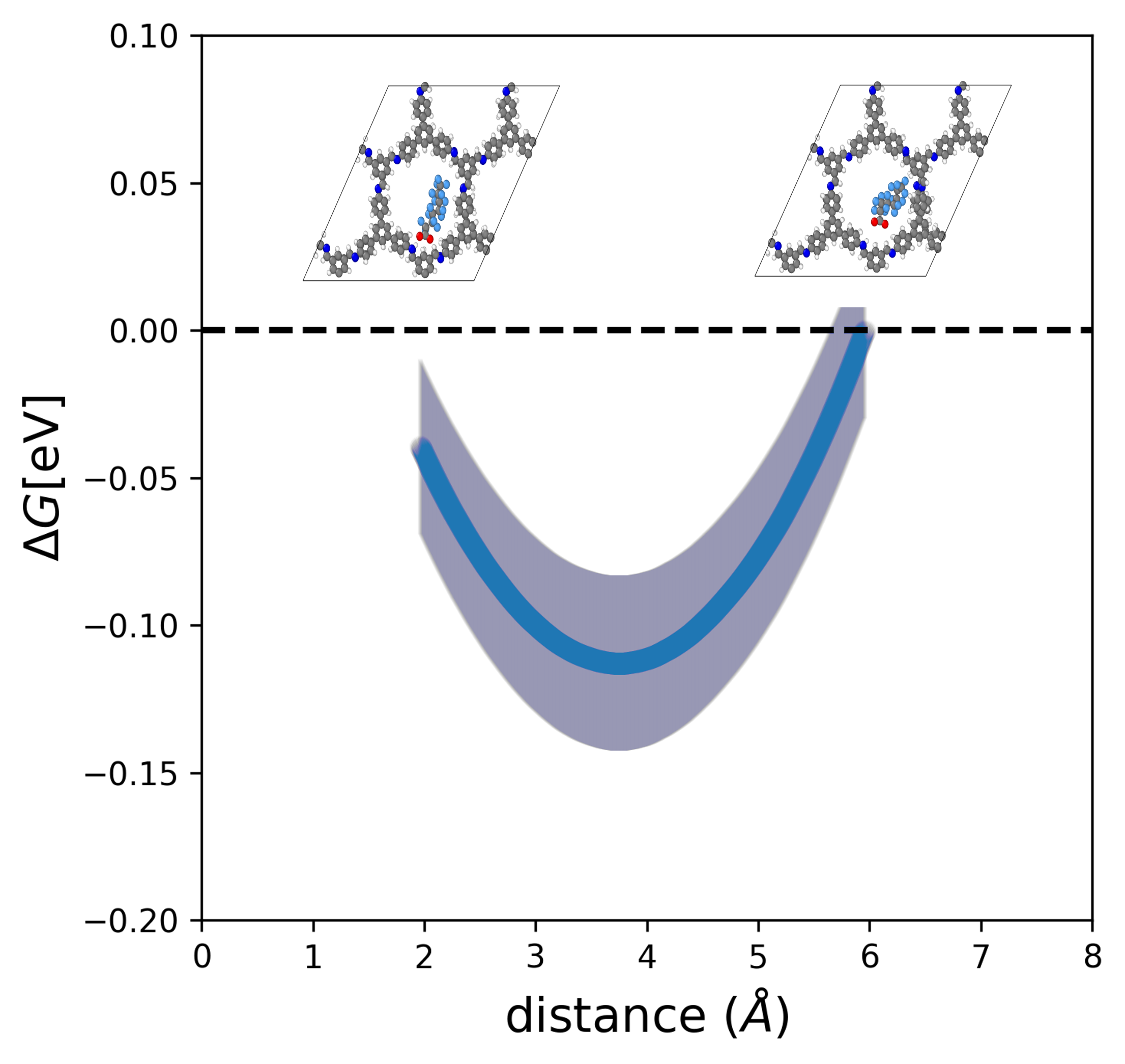


**Figure S17:** Free energy change (ΔG) as a function of distance of the virtual spring connecting the protonated nitrogen atom and the anionic headgroup of PFOA (red spheres). The shaded area indicates the standard deviation over the 5 simulations. Structure visualization via Ovito.^[15]^ All simulation files are publicly available at <https://github.com/JaGeo/TAPB-TFB-COF-PFAS> (archived in https://doi.org/10.5281/zenodo.16560902).

**S8 References**

[1] X. Kong, Z. Wu, M. Strømme, C. Xu, *J. Am. Chem. Soc.* **2024**, *146*, 742–751.

[2] I. R. Speight, K. J. Ardila-Fierro, J. G. Hernández, F. Emmerling, A. A. L. Michalchuk, F. García, E. Colacino, J. Mack, *Nat. Rev. Methods Primer* **2025**, *5*, DOI 10.1038/s43586-025-00401-2.

[3] I. Batatia, P. Benner, Y. Chiang, A. M. Elena, D. P. Kovács, J. Riebesell, X. R. Advincula, M. Asta, M. Avaylon, W. J. Baldwin, F. Berger, N. Bernstein, A. Bhowmik, S. M. Blau, V. Cărare, J. P. Darby, S. De, F. Della Pia, V. L. Deringer, R. Elijošius, Z. El-Machachi, F. Falcioni, E. Fako, A. C. Ferrari, A. Genreith-Schriever, J. George, R. E. A. Goodall, C. P. Grey, P. Grigorev, S. Han, W. Handley, H. H. Heenen, K. Hermansson, C. Holm, J. Jaafar, S. Hofmann, K. S. Jakob, H. Jung, V. Kapil, A. D. Kaplan, N. Karimitari, J. R. Kermode, N. Kroupa, J. Kullgren, M. C. Kuner, D. Kuryla, G. Liepuoniute, J. T. Margraf, I.-B. Magdău, A. Michaelides, J. H. Moore, A. A. Naik, S. P. Niblett, S. W. Norwood, N. O’Neill, C. Ortner, K. A. Persson, K. Reuter, A. S. Rosen, L. L. Schaaf, C. Schran, B. X. Shi, E. Sivonxay, T. K. Stenczel, V. Svahn, C. Sutton, T. D. Swinburne, J. Tilly, C. van der Oord, E. Varga-Umbrich, T. Vegge, M. Vondrák, Y. Wang, W. C. Witt, F. Zills, G. Csányi, **2024**, arXiv preprint, DOI: 10.48550/ARXIV.2401.00096.

[4] A. Jain, S. P. Ong, G. Hautier, W. Chen, W. D. Richards, S. Dacek, S. Cholia, D. Gunter, D. Skinner, G. Ceder, K. A. Persson, *APL Mater.* **2013**, *1*, 011002.

[5] M. K. Horton, P. Huck, R. X. Yang, J. M. Munro, S. Dwaraknath, A. M. Ganose, R. S. Kingsbury, M. Wen, J. X. Shen, T. S. Mathis, A. D. Kaplan, K. Berket, J. Riebesell, J. George, A. S. Rosen, E. W. C. Spotte-Smith, M. J. McDermott, O. A. Cohen, A. Dunn, M. C. Kuner, G.-M. Rignanese, G. Petretto, D. Waroquiers, S. M. Griffin, J. B. Neaton, D. C. Chrzan, M. Asta, G. Hautier, S. Cholia, G. Ceder, S. P. Ong, A. Jain, K. A. Persson, *Nat. Mater.* **2025**, DOI 10.1038/s41563-025-02272-0.

[6] J. Schmidt, N. Hoffmann, H. Wang, P. Borlido, P. J. M. A. Carriço, T. F. T. Cerqueira, S. Botti, M. A. L. Marques, *Adv. Mater.* **2023**, *35*, 2210788.

[7] S. Park, K. Schulten, *J. Chem. Phys.* **2004**, *120*, 5946–5961.

[8] C. Jarzynski, *Phys. Rev. Lett.* **1997**, *78*, 2690–2693.

[9] A. P. Thompson, H. M. Aktulga, R. Berger, D. S. Bolintineanu, W. M. Brown, P. S. Crozier, P. J. In ’T Veld, A. Kohlmeyer, S. G. Moore, T. D. Nguyen, R. Shan, M. J. Stevens, J. Tranchida, C. Trott, S. J. Plimpton, *Comput. Phys. Commun.* **2022**, *271*, 108171.

[10] D. P. Kovács, J. H. Moore, N. J. Browning, I. Batatia, J. T. Horton, Y. Pu, V. Kapil, W. C. Witt, I.-B. Magdău, D. J. Cole, G. Csányi, *J. Am. Chem. Soc.* **2025**, *147*, 17598–17611.

[11] D. Rappoport, F. Furche, *J. Chem. Phys.* **2010**, *133*, 134105.

[12] F. Weigend, R. Ahlrichs, *Phys. Chem. Chem. Phys.* **2005**, *7*, 3297.

[13] N. Mardirossian, M. Head-Gordon, *J. Chem. Phys.* **2016**, *144*, 214110.

[14] A. Najibi, L. Goerigk, *J. Chem. Theory Comput.* **2018**, *14*, 5725–5738.

[15] A. Stukowski, *Model. Simul. Mater. Sci. Eng.* **2010**, *18*, 015012.
